# Study protocol for a randomized controlled trial of a self-determination theory–based psychoeducational intervention to enhance autonomous motivation for exercise in female university students

**DOI:** 10.1371/journal.pone.0355374

**Published:** 2026-08-07

**Authors:** Sonia García-Merino, Patricia Ruiz-Bravo, Germán Díaz Ureña

**Affiliations:** 1 Institute of Health and Sport Sciences, Faculty of Health Science, Universidad Francisco de Vitoria, Madrid, Spain; 2 Faculty of Health Science, Universidad Francisco de Vitoria, Madrid, Spain; Soochow University, CHINA

## Abstract

Physical inactivity, maladaptive exercise motives, and body image concerns are highly prevalent among young adult women and are associated with reduced psychological well-being and increased risk for disordered eating. Although previous interventions have addressed physical activity or body image separately, few have simultaneously targeted motivational quality and body-related perceptions within an integrated theoretical framework. This study protocol describes a randomized controlled trial designed to evaluate the effectiveness of a psychoeducational intervention based on Self-Determination Theory and using functionality-oriented body image strategies. The aim is to enhance autonomous motivation for exercise and improve psychological well-being among female university students in Health Sciences. Participants aged 18–25 years will be randomly allocated (1:1) to an experimental group or a wait-list control group following baseline assessment. Outcome measures will be collected at baseline (T0), post-intervention (T1), and six-month follow-up (T2). The intervention consists of six face-to-face group sessions that include autonomy-supportive strategies, competence-building activities, and functionality-based body image content emphasizing body capabilities rather than appearance. The primary outcome is autonomous motivation for exercise, assessed using the Behavioral Regulation in Exercise Questionnaire-3 and operationalized through the Relative Autonomy Index at six-month follow-up. Secondary outcomes include satisfaction and frustration of basic psychological needs, physical activity levels, positive body image, and risk of eating disorders. Data will be analyzed using mixed model for repeated measures under an intention-to-treat framework to account for repeated measures and missing data. Mediation analyses will examine whether changes in autonomous motivation explain the effects of the intervention on physical activity, positive body image, and eating disorder risk. This study will provide evidence on the effectiveness of an integrated, theory-based intervention targeting motivation and body image in young adult women. The trial is registered at ClinicalTrials.gov (Identifier: NCT07506343).

## Introduction

The university period represents a developmental stage characterized by increased autonomy, profound lifestyle changes, and heightened vulnerability to health‑related behavior disruptions. Insufficient physical activity (PA) and high sedentary time remain highly prevalent among university students worldwide [1]. Global surveillance data show a persistent gender gap in PA participation: women report markedly higher inactivity rates (31.7%) compared to men (23.4%), reaching up to 42.3% in high‑income Western countries [2]. These patterns are concerning, as physical inactivity tends to increase during early adulthood and is associated with adverse long‑term physical and psychological outcomes [3,4].

Concurrently, the university stage coincides with elevated susceptibility to body dissatisfaction, internalization of appearance ideals, and risk factors for eating disorders (ED). Recent epidemiological data in Spain report a point prevalence of 7.8% for EDs, with young women representing the group at highest risk [5]. Body dissatisfaction, exposure to unrealistic appearance ideals, and the pervasive influence of social media amplify this vulnerability [6,7]. Importantly, PA in this population can become maladaptive when driven by controlled motives such as guilt, social pressure, or appearance management [8,9]. Understanding not only whether, but why, young women engage in PA is therefore essential to prevent maladaptive patterns and promote sustained well‑being.

Self-Determination Theory (SDT) provides a comprehensive framework for understanding motivational processes associated with PA engagement and psychological health [10,11]. SDT differentiates autonomous motivation, rooted in personal values, interest, and volition, from controlled motivation, which arises from internal or external pressures. Autonomous motivation predicts sustained PA, greater psychological well-being, and healthier body attitudes, whereas controlled motivation is associated with poorer adherence and emotional distress [12,13]. Central to SDT is the satisfaction of the basic psychological needs for autonomy, competence, and relatedness; meta-analytic evidence shows that interventions supporting these needs improve motivational quality and promote health-related behavioral regulation [14,15]. Recent analyses further demonstrate that combining multiple need-supportive strategies, autonomy-supportive communication, structure, and relational support produces the strongest effects on motivation and PA adherence [14].

In parallel, increasing evidence emphasizes the relevance of positive body image, particularly functionality appreciation, the recognition of what the body can do rather than how it looks, as a protective factor for young women [16,17]. Functionality-focused interventions have shown significant improvements in body appreciation and reductions in ED risk indicators among adolescents and young women [16,18]. This approach aligns conceptually with SDT, as it shifts individuals toward intrinsically rewarding, self-endorsed reasons for engaging in PA. Integrating functionality appreciation into motivational interventions may reduce maladaptive appearance-based motives while simultaneously reinforcing autonomous regulation and intrinsic reasons for engaging in PA.

Despite advances in both research areas, notable gaps remain. Few university‑based interventions jointly target (a) the quality of motivation for PA and (b) body image or ED‑risk factors within a unified SDT‑informed framework. Existing interventions often address these components separately, limiting their effectiveness. Moreover, evidence from Mediterranean populations is scarce, and many studies lack follow-up assessments or include only short-term evaluations, restricting conclusions regarding the sustainability of observed changes [15,19].

To address these gaps, the present study describes a randomized controlled trial evaluating a psychoeducational intervention designed to enhance autonomous motivation for PA and promote positive body image among female Health Sciences students. Grounded in SDT and enriched with functionality-focused components, the intervention targets theoretically central mechanisms, basic psychological need satisfaction and autonomous regulation, to support sustainable PA engagement and reduce vulnerability to ED-related risk factors. By intervening in a high-risk developmental period and focusing on future health professionals, this trial holds potential not only for improving individual well-being but also for fostering autonomy-supportive communication styles that may influence future clinical practice.

Based on the theoretical framework, existing empirical evidence, and the identified gaps in the literature, the present study proposes the following hypotheses:

*Primary hypothesis*

H1. The intervention will significantly increase autonomous PA motivation (BREQ-3; Relative Autonomy Index) at post-intervention (T1) and follow-up (T2).

*Secondary hypotheses*

H2. Participants will report higher basic psychological need satisfaction (autonomy, competence, and relatedness; BPNSFS) at post-intervention (T1) and follow-up (T2).
H3. PA levels (IPAQ-SF) will increase significantly at T1 and T2.
H4. Participants will show significant improvements in positive body image (BAS-2).
H5. Eating disorder risk (EAT-26) will be significantly reduced.

*Mediation hypothesis (key mechanism)*

H6. Changes in autonomous motivation will mediate the intervention’s effect on PA levels and health-related psychological outcomes.

## Materials and methods

This study protocol follows the SPIRIT (Standard Protocol Items: Recommendations for Interventional Trials) 2025 guidelines (see supplementary material S1 File) [20]. The intervention is described in accordance with the Template for Intervention Description and Replication checklist (see supplementary material S2 File) [21], which complements SPIRIT 2025 recommendations for reporting interventions.

### Study design

This study design is a randomized controlled trial (RCT), comprising an experimental group and a control group. Three assessment time points are planned: baseline (T0), immediate post-intervention (T1), and a six-month follow-up after completion of the intervention (T2). This design will allow examination of the effectiveness of a psychoeducational intervention grounded in Self-Determination Theory [10], aimed at fostering more autonomous motivation toward physical activity among female university students.

The study will be conducted at Universidad Francisco de Vitoria (Madrid, Spain), in facilities designated for face-to-face group workshops. The total duration of the project is estimated at twelve months, including preparation, recruitment, intervention implementation, data analysis phases, and dissemination of results.

### Participants’ eligibility

The target population will consist of female university students aged 18–25 years, enrolled in Health Sciences degree programs. This age range was selected because it corresponds to a developmental stage characterized by significant changes in health-related behaviors and increased psychological vulnerability, thus highlighting the relevance of preventive interventions within the university context [4,22].

The focus on Health Sciences students is particularly relevant, as they are future health professionals. Research suggests a ‘motivational spill-over’ effect, where fostering autonomous regulation in their own health behaviors (like PA) can later improve their professional ability to support their patients’ autonomy [12].

Participants must meet all of the following criteria:

- Female sex
- Aged between 18 and 25 years
- Enrolled in a Health Sciences degree program
- Provide written informed consent indicating voluntary participation

Participants will be excluded if they:

- Report a current or previous diagnosis of an eating disorder

### Recruitment

Participant recruitment will be conducted centrally by the university to ensure compliance with institutional data protection regulations. This communication will include standardized information about the study, prepared and authorized by the Ethics Committee, as well as a link to the online registration form. Students who are interested may voluntarily access the link, review detailed information about the study, and complete the eligibility screening. Because all eligible students receive a personal email invitation (a census of the eligible population), no probability sub-sampling of the invitation frame is applied. To characterize and address volunteer self-selection at the response stage, the overall and stratum-specific response rate will be reported, respondents will be compared with the full invited eligible population on available administrative characteristics (degree program and year of study), and a post-stratification / inverse-probability-of-response weighting sensitivity analysis will be conducted (see supplementary material S3 File). Only those who self‑enroll and meet the inclusion criteria will be asked to provide written informed consent prior to participation. At no stage during this initial outreach will the research team access personal data of non‑interested students, thereby ensuring full adherence to data protection guidelines and safeguarding confidentiality throughout the recruitment process. No financial compensation will be provided for participation. However, participants will be offered access to a structured psychoeducational program and, in the case of the control group, delayed access to the full intervention after completion of the study. This approach was selected to balance ethical considerations and feasibility within the academic context.

### Withdrawals

Participants will be free to withdraw from the study at any time without academic or personal consequences. Participants will also be withdrawn if a current or previous diagnosis of an eating disorder is identified after the initial screening phase. In such cases, participants will be debriefed and referred to specialized university health services. All withdrawals will be fully documented, including the reason for withdrawal and the time point at which it occurs. Data analyses will be conducted according to the intention-to-treat principle, incorporating all available data up to the time of withdrawal in order to preserve the benefits of randomization and minimize attrition bias.

### Sample size

Sample size was initially estimated using a repeated-measures ANOVA framework as an approximation of the planned longitudinal analysis, implemented in G*Power software (version 3.1.9.6). A small effect size was assumed (f = 0.10), consistent with meta-analytic evidence from self-determination theory–based interventions (g ≈ 0.40) [14] and body image programs (g ≈ 0.40) [19].

With a significance level of α = 0.05 and statistical power (1 − β) of 0.80, and assuming two groups with three repeated measurements, the minimum required sample size was estimated to be 164 participants (82 per group). To account for an anticipated attrition rate of approximately 20%, the target sample size was increased to 196 participants.

Given the longitudinal design of the study and the use of mixed models for repeated measures (MMRM) as the primary analytical approach, simulation-based power analyses were subsequently conducted in R to obtain more realistic estimates under plausible assumptions.

These simulations incorporated baseline adjustment, within-subject correlation, and missing data patterns consistent with behavioral interventions. Effect sizes were specified in the small-to-moderate range (d ≈ 0.20–0.30), and assumptions regarding residual variability and within-subject correlation were informed by prior literature on autonomous motivation (RAI).

Power was evaluated with respect to the primary contrast at six-month follow-up time point.

Simulation results indicated that the planned sample size provides moderate, and in some scenarios approaching adequate, statistical power under realistic assumptions, while reflecting the inherent variability associated with behavioral and psychological outcomes.

These complementary approaches ensured both consistency with conventional sample size estimation methods and alignment with the planned analytical model.

To account for potential deviations from the target sample size, contingency strategies were pre-specified. If the final sample exceeds the planned size, all participants will be retained in the analyses to maximize statistical power and precision. If the achieved sample size falls below the target, additional recruitment efforts will be undertaken following the same procedures. Based on the initial assumptions, a minimum sample size of 70 participants per group would still provide adequate statistical power (1 − β = 0.80) to detect moderate effects (g ≈ 0.40).

Detailed simulation parameters are provided in the Statistical Analysis Plan.

### Randomization, blinding, and allocation

Following baseline assessment, participants will be randomly allocated to either the experimental or the control group in a 1:1 ratio. The allocation sequence will be generated in R using the *blockrand* package by an independent researcher not involved in participant recruitment, intervention delivery or outcome assessment, with a random seed kept confidential until recruitment is complete.

Block randomization will be used, with permuted blocks of variable size (4, 8, and 12) and 1:1 allocation within each block, without stratification given the homogeneity of the target population in age and academic context. Using variable block sizes that are not disclosed to enrolling staff preserves allocation concealment by preventing prediction of forthcoming assignments, while ensuring balanced group sizes throughout recruitment and minimizing selection bias.

Recruiting and enrolling staff remain masked to the allocation sequence and to the (variable) block sizes throughout the trial. As a post-hoc verification of the absence of allocation selection bias, a Berger–Exner test will be applied to check for any association between the allocation sequence and baseline prognostic characteristics.

Due to the nature of the psychoeducational intervention, blinding of participants and intervention providers (facilitators) will not be feasible. Outcome data will be collected using self-administered standardized questionnaires, minimizing assessor-related bias.

Researchers involved in data management and statistical analysis will remain blinded to group allocation until the primary analyses are completed. Data will be coded using anonymized group identifiers to ensure concealment during analysis.

This approach ensures appropriate separation between sequence generation, allocation concealment, and blinding procedures, in accordance with established guidelines for randomized controlled trials.

### Procedure and intervention

The intervention is grounded in Self-Determination Theory [10,11] and is described following TIDieR recommendations to ensure clarity and reproducibility. It adopts an autonomy-supportive approach, integrating strategies aimed at satisfying the basic psychological needs of autonomy, competence, and relatedness. This approach has consistently been associated with increases in autonomous motivation and improvements in health-related behaviors in educational and physical activity contexts [14,15,23].

After providing written informed consent, participants will complete the baseline assessment using self-administered digital questionnaires designed to evaluate motivation toward physical activity and secondary psychological and behavioral study variables. Upon completion of the initial assessment, participants will be randomly assigned in a 1:1 ratio to either the experimental group or the control group.

#### Experimental intervention.

Participants assigned to the experimental group will take part in a face-to-face psychoeducational program consisting of six 60-minute group workshops, conducted in small groups of up to 15 participants. Each participant remains in the same delivery subgroup across the six sessions. This format facilitates social interaction and the creation of a need-supportive climate, factors identified as relevant in interventions based on Self-Determination Theory (SDT). Sessions are facilitated by trained professionals with experience in exercise science and psychonutrition, who receive standardized training prior to intervention delivery to ensure consistency across groups. Facilitators complete standardized training covering SDT principles, functionality‑based body image strategies, session structure, and fidelity procedures. The intervention includes structured presentation slides, participant worksheets, guided experiential activities, and reflective exercises. Detailed intervention materials are available from the authors upon reasonable request. Minor contextual adaptations (e.g., examples or wording adjustments) may be made, but core intervention components remain unchanged. The intervention will be delivered following a standardized manual based on established SDT techniques, including autonomy support, structure, and involvement [13,14], as summarized in Table 1.

**Table 1:** Structure, objectives, and Basic Psychological Needs-Supportive Strategies targeted in the intervention workshops.

Workshop	Primary focus	Need-Supportive Strategies (SDT-based)	Basic Psychological Needs targeted
1	Awareness of motivational quality toward PA	Providing a meaningful rationale: explaining the “why” behind PA engagement.	Autonomy
2	Personal values and autonomously chosen goals	Autonomy support: promoting the internalization of PA goals based on personal interests.	Autonomy & Competence
3	Perceived competence and flexible planning	Structure: providing clear guidance, mastery-oriented feedback, and skill-building for PA.	Autonomy & Competence
4	Positive body image from a functionality-oriented perspective	Acknowledging perspectives: validating participants’ feelings and fostering intrinsic values.	Autonomy & Relatedness
5	Social support and supportive relational climate	Involvement: fostering a warm, supportive, and non-judgmental climate for PA.	Relatedness
6	Maintenance of change and management of difficulties	Action planning: identifying future supports and strategies for managing PA challenges.	Competence

**Note:** SDT = Self-Determination Theory; PA = Physical Activity.

Each workshop combines brief psychoeducational content with interactive, experiential activities designed to promote reflection, personal meaning, and supportive peer interaction. Activities are structured to support autonomy (choice provision, validation of personal perspectives), competence (mastery‑oriented tasks, individualized progress‑focused feedback), and relatedness (collaborative group climate). This multicomponent structure aligns with evidence identifying these elements as key mechanisms for enhancing autonomous motivation in health‑behavior interventions [14,15].

A distinctive feature of the program is the incorporation of functionality‑oriented body image content. This approach shifts attention from appearance to bodily capabilities and has been associated with improvements in body appreciation and reductions in ED‑related risk factors among young women [16–18]. Integrating functionality appreciation within an SDT‑based framework is expected to reduce appearance‑based exercise motives and reinforce autonomous regulation, helping prevent maladaptive patterns such as compulsive or guilt‑driven exercise [8,9].

Across the six workshops (Table 1), participants will progressively work on: (1) awareness of motivational quality toward exercise; (2) identification of personal values and self‑endorsed goals; (3) competence development through flexible planning; (4) functionality‑focused body appreciation; (5) strengthening social support; and (6) maintenance of behavior change and management of challenges. Fidelity will be monitored using session checklists completed after each workshop. This design integrates components targeting motivational and psychosocial processes identified as relevant factors in the prevention and reduction of eating disorder symptomatology and other health-related behaviors [19,24]. Participants in both groups will be asked to maintain their usual lifestyle habits throughout the study period, and no restrictions will be imposed on concurrent physical activity or health-related behaviors.

Assessments will be conducted at three time points: baseline, immediate post-intervention, and six-month follow-up. To ensure both adherence and intervention fidelity, facilitators will receive prior specific training and follow a structured session guide. Fidelity to the protocol will be monitored by the research team using standardized checklists completed after each workshop. Additionally, a set of retention strategies will be implemented to minimize attrition and missing data, including periodic email reminders and advance notifications of session schedules. Attendance will be systematically monitored throughout the intervention, and participants will be reminded of the importance of continuous participation from the outset to ensure data completeness across all assessment points.

Table 2 presents the standardized pedagogical structure applied in each workshop session, comprising six sequential phases that combine initial welcome, experiential activation, psychoeducational input, group-based activities, individual reflection, and a brief closing synthesis.

**Table 2:** Standard pedagogical structure of each workshop session.

Session phase	Duration	Description
Welcome and psychological climate	5 min	Facilitators welcome participants, present the objectives of the session, and establish a supportive and non-evaluative group climate
Experiential activation	10 min	Short reflective or experiential activity designed to activate participants’ previous experiences and beliefs related to the workshop topic
Psychoeducational content	15 min	Brief evidence-based explanation of the key concept addressed in the workshop (e.g., motivational regulation, body functionality, social support)
Experiential group activity	20 min	Interactive activities conducted in pairs or small groups allowing participants to apply the concepts through discussion, reflection, or body-awareness exercises
Personal reflection and transfer	5 min	Individual reflection on how the workshop content relates to participants’ own behaviors and experiences
Closing and synthesis	5 min	Summary of key ideas and encouragement to apply the concepts in everyday life

### Wait-list control condition

Participants assigned to the control group will remain on the wait-list during the intervention and follow-up periods and will not receive any specific intervention. To ensure intervention equity, they will be offered access to the full program upon completion of the T2 assessment.

Participants in both groups will be allowed to continue their usual activities and access external health-related services (e.g., psychological support, personal training, or dietary counseling) throughout the study period. To monitor potential sources of contamination, participants will be asked to report any engagement in such services during the study.

These data will be recorded and considered in the interpretation of the findings. If deemed necessary, additional sensitivity analyses will be conducted to explore the potential impact of external interventions on the study outcomes.

All protocol deviations, including non-adherence to the intervention or engagement in external programs, will be documented and reported following established guidelines for randomized controlled trials.

### Measures

Assessments will be conducted at three time points: baseline, post-intervention, and six-month follow-up.

### Primary outcome

The primary outcome of the study will be between-group differences in autonomous motivation toward physical activity at six-month follow-up, assessed using the Behavioral Regulation in Exercise Questionnaire–3 (BREQ-3) [25], validated in the Spanish population [26]. The instrument consists of 23 items distributed across six subscales representing different forms of motivational regulation: amotivation, external regulation, introjected regulation, identified regulation, integrated regulation, and intrinsic motivation. Responses are recorded on a five-point Likert scale ranging from 0 (“Not true for me”) to 4 (“Very true for me”).

From these scores, the Relative Autonomy Index (RAI) will be computed to quantify the overall degree of self-determination in exercise motivation. Higher RAI scores indicate more autonomous forms of motivation, whereas lower scores reflect a predominance of controlled regulation or amotivation. Additionally, following recent meta-analytic recommendations, scores for each of the six subscales will be reported independently to avoid masking potential distinct effects of different regulatory styles on physical activity outcomes [25,27]. The scale has demonstrated a quasi-simplex pattern of associations and high temporal stability in Spanish samples [26].

### Secondary outcomes

Secondary outcomes include psychological and behavioral variables relevant to understanding motivational change. Satisfaction and frustration of basic psychological needs will be assessed using the Basic Psychological Need Satisfaction and Frustration Scale (BPNSFS) [28], a 24-item instrument measuring autonomy, competence, and relatedness, each examined in terms of both satisfaction and frustration dimensions.

Physical activity levels will be measured using the short form of the International Physical Activity Questionnaire (IPAQ-short) [29], which estimates time spent in vigorous and moderate physical activity as well as walking, expressed in MET-minutes per week. Because the BREQ‑3 specifically assesses exercise motivational regulations and the IPAQ‑SF captures general physical activity, both instruments will be used to comprehensively reflect behavioral engagement and its motivational determinants.

Positive body image will be assessed using the Body Appreciation Scale–2 (BAS-2) [30], Spanish adaptation [31], a 10-item Likert-type scale designed to evaluate body acceptance and respect.

Finally, risk of eating disorders will be measured using the Eating Attitudes Test–26 (EAT-26) [32], a widely used instrument in epidemiological and preventive research.

### Administration procedure

Questionnaires will be administered in a predefined order designed to minimize response bias and safeguard participant well-being: first the BPNSFS, followed by the BREQ-3, the IPAQ-short, the BAS-2, and finally the EAT-26, given its more sensitive nature. The estimated completion time for the full assessment battery is approximately 25–35 minutes.

All assessments will be conducted in digital format to ensure accessibility, standardized administration, and data confidentiality.

### Data management

All data collected during the study will be stored on a secure, access‑restricted server hosted by the University. Each participant will be assigned a unique identification code, and all datasets used for analysis will be coded to ensure participant anonymity by removing any direct or indirect identifiers. The correspondence between participant identities and identification codes will be kept in an encrypted, password‑protected file accessible only to the principal investigators. Data entry will be conducted electronically through a secure platform, and regular data quality checks will be performed to detect inconsistencies or missing values. Access to the raw data during the study will be limited to authorized members of the research team who have completed institutional training in data protection and confidentiality. All data management procedures will comply with the European Union General Data Protection Regulation [33] and institutional policies on data protection and research integrity.

Missing data will be handled under an intention-to-treat framework using likelihood-based mixed models for repeated measures (MMRM). This approach incorporates all available observed data without the need for prior imputation and provides valid inferences under the assumption that data are missing at random (MAR). Participants will contribute data up to the point at which observations are available, and no ad hoc imputation procedures will be applied. All datasets and accompanying metadata will be retained for a minimum of five years after publication to allow verification, auditing, or secondary analyses.

### Statistical analysis

All statistical analyses will be pre-specified prior to database lock and conducted using RStudio (v.2026.01.1 + 403, Posit Software). Analyses will follow the intention-to-treat principle, as the primary analytical approach. In addition, per-protocol analyses will be conducted as sensitivity analyses to examine the robustness of findings and to account for potential variations in intervention fidelity (e.g., adherence to the full set of sessions). Descriptive statistics will first be calculated for all study variables, including measures of central tendency and dispersion. Assumptions of normality will be assessed using appropriate statistical tests and graphical methods.

To evaluate the effect of the intervention on the primary outcome, a mixed model for repeated measures (MMRM) will be used, including all post-randomization assessments of autonomous motivation toward physical activity (BREQ-3), Fixed effects will include treatment group (experimental vs. control), assessment time, the treatment-by-time interaction, and baseline values of the outcome as a covariate.

An appropriate covariance structure (e.g., unstructured or alternative based on model fit indices) will be specified to account for within-participant correlations across repeated measurements. The primary endpoint will be the between-group difference at six-month follow-up, while effects at post-intervention will be considered secondary analyses. Least-squares mean differences between groups will be estimated from the model, along with corresponding 95% confidence intervals and p-values. This likelihood-based approach uses all available observed data without imputing missing values and provides valid inferences under the missing at random (MAR) assumption. Secondary outcomes, including satisfaction and frustration of basic psychological needs, physical activity levels, positive body image, and risk of eating disorders, will be analyzed using equivalent MMRM approaches. In cases where parametric assumptions are violated, robust mixed-effects models and bootstrapping procedures will be applied to obtain reliable parameter estimates. Exploratory analyses of the six BREQ‑3 subscales will follow the same mixed‑effects modelling approach. In addition, mediation will be tested with a half-longitudinal model [34]: group is the independent variable, the change in autonomous motivation (BREQ-3 RAI) from T0 to T1 is the mediator, and the changes in physical activity (IPAQ-SF), positive body image (BAS-2), and eating disorder risk (EAT-26) from T1 to T2 are the outcomes. The primary specification is a single-level structural equation model (lavaan) with bias-corrected bootstrap confidence intervals for the indirect effect; a multilevel SEM that accounts for the partially nested delivery subgroups is reported as a sensitivity analysis. Full detail is given in the SAP (see Supporting information S3 File). Statistical significance will be set at p < .05. Effect sizes will be calculated to facilitate interpretation of the magnitude of effects. Ninety-five percent confidence intervals will be reported for all relevant estimates.

To enhance transparency and reproducibility, a complete Statistical Analysis Plan (SAP, v2.0), structured following the template by [35], is provided as supplementary material (S3 File).

### Ethics and dissemination

All collected data will be coded through the assignment of a unique identification code to each participant, thereby preventing the inclusion of personally identifiable information in the analytical datasets. The correspondence between identification codes and personal data will be stored in a separate, password-protected file on a secure server. Data management procedures will comply with the European Union General Data Protection Regulation [33]. Given the low-risk nature of the psychoeducational intervention, no independent Data Monitoring Committee has been established. Study oversight will be conducted by the research team in accordance with ethical approval and institutional guidelines. No formal auditing procedures are planned for this trial, as it involves minimal risk and is conducted within a single institutional setting.

Questionnaires will be administered in digital format through a secure platform with restricted access limited to authorized members of the research team. Upon completion of the study, coded data will be archived in an institutional repository to ensure long-term preservation and availability for verification and secondary analyses, in accordance with the FAIR principles (Findable, Accessible, Interoperable, and Reusable).

The study was approved by the Research Ethics Committee of Universidad Francisco de Vitoria (reference number: 29/2026) and is registered at ClinicalTrials.gov under the identifier NCT07506343. All participants will receive detailed written information regarding the study objectives, procedures, potential risks, and anticipated benefits, and will provide written informed consent before participation. Participants will retain the right to withdraw from the study at any time without academic or personal consequences. Any important modifications to the study protocol (e.g., changes to eligibility criteria, outcomes, or analysis plan) will be submitted for approval to the Research Ethics Committee of Universidad Francisco de Vitoria prior to implementation. Approved amendments will be updated in the trial registry (ClinicalTrials.gov) and communicated to all members of the research team. When relevant, participants will be informed of any changes that may affect their participation, and they will be provided with information regarding the study results, communicated using terminology that is appropriate and comprehensible.

No physical risks are anticipated as a result of participation in the intervention. However, given the inclusion of sensitive questionnaires related to body image and eating disorder risk, a participant well-being protocol will be implemented. This protocol includes referral to university psychological support services if risk indicators are identified. No financial compensation will be provided; however, equity will be ensured by offering participants in the control group full access to the intervention program after completion of the follow-up assessment.

The wait-list control design was selected for both methodological and ethical reasons. From an ethical perspective, it ensures that all participants will eventually have access to the intervention, which is particularly relevant in university-based preventive programs targeting body image and motivational processes. Methodologically, this design may enhance participant retention and reduce attrition bias. Moreover, the use of a wait-list rather than an active or minimal-intervention control condition allows clearer detection of intervention-specific effects on perceived autonomy support and motivational regulation.

### Study and participant timeline

The study is planned to be conducted over an estimated twelve-month period. At the time of this submission, the project is in its preparatory phase; no participant recruitment has begun, and no data have been generated. Participant recruitment is expected to commence in October 2026 and to be completed by December 2026. Baseline data collection will be conducted in January 2027. The six-session group intervention will then be delivered over the subsequent weeks. Immediate post-intervention assessments are expected to be completed by March 2027, following completion of the intervention. After a planned six-month interval, follow-up data collection is anticipated to take place in September 2027. Statistical analyses and final results are expected to be completed and ready for dissemination between October and November 2027. This timeline has been designed to ensure feasibility and full compliance with institutional and journal requirements.

**Fig 1:**
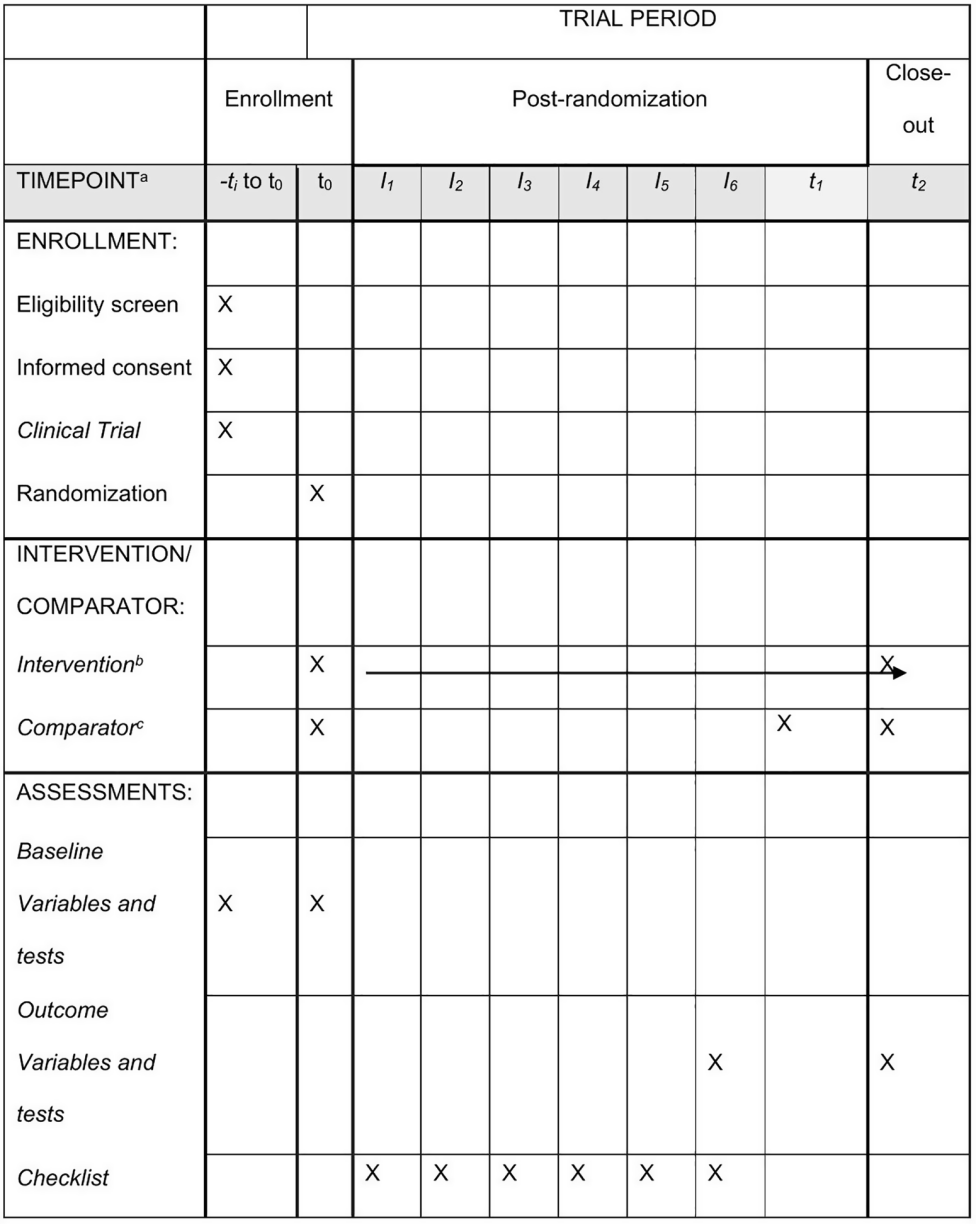
Participant timeline: Schedule of enrollment, interventions, and assessments. ^a^ Timepoints correspond to baseline (T0), immediate post‑intervention (T1), and six‑month follow‑up (T2). No additional time windows are required because all assessments are scheduled within fixed, predefined periods. ^b^ The intervention is delivered across six structured weekly group workshops; therefore, the arrow does not indicate uninterrupted delivery but rather a multi‑session structured psychoeducational program. ^c^ The control group does not receive any active intervention during the trial period and will be offered access to the full program only after completing the follow‑up assessments at T2.

The participant timeline is presented in accordance with SPIRIT 2025 recommendations and illustrates the schedule of enrollment, allocation, intervention delivery, and outcome assessments for the trial [20] (Fig 1).

## Discussion

This study protocol outlines a randomized controlled trial evaluating a psychoeducational intervention that integrates Self-Determination Theory (SDT) principles and functionality-oriented body image strategies to promote autonomous PA engagement and psychological well-being among female university students. By addressing motivational quality, PA behavior, and psychosocial risk factors concurrently, the intervention targets mechanisms identified as central to sustainable behavior change and ED prevention [13,19].

The urgency of developing preventive interventions in this population is supported by epidemiological evidence. In Spain, the point prevalence of EDs among young women has been estimated at 7.8% [5]. Using the “iceberg metaphor,” these authors highlight that the observable eating behaviors represent only the visible portion of a deeper psychological process characterized by body dissatisfaction and personal disqualification. Within this context, the university environment represents a particularly valuable setting for prevention because it provides a communal and academic atmosphere where health promotion programs can be integrated into students’ educational experiences [36]. Consequently, interventions delivered in university contexts may offer an efficient strategy to address early psychosocial risk factors associated with disordered eating and maladaptive exercise behaviors.

The relevance of this approach is further supported by prior evidence demonstrating that SDT‑based interventions can produce small‑to‑moderate improvements in perceived autonomy support, autonomy, competence, and motivation [14]. Comparable patterns have been observed in organized PA settings, where satisfaction of basic psychological needs predicts greater enjoyment, persistence, and adaptive regulatory profiles [27]. Similarly, research in educational contexts shows that autonomy‑supportive strategies yield larger effects on motivational quality, underscoring the value of integrating multiple need‑supportive techniques within complex interventions [23]. Collectively, these findings reinforce the theoretical and practical justification for a SDT-informed program such as the one outlined in this protocol. Within this framework, improvements in PA engagement are expected to occur through increased satisfaction of psychological needs and the progressive internalization of exercise-related motives. In line with the proposed mediation hypothesis, autonomous motivation may function as a key psychological mechanism linking need-supportive intervention components with improvements in PA engagement and related health outcomes.

A major strength of the protocol is its strong and explicit theoretical grounding. SDT provides a comprehensive framework for understanding how the satisfaction of autonomy, competence, and relatedness needs fosters higher-quality motivation and adaptive behavioral regulation [10,11]. The intervention incorporates several motivation and behavior change techniques identified through expert consensus, including autonomy‑supportive communication, the provision of meaningful rationales, opportunities for individual choice, competence‑enhancing activities, and the creation of supportive social climates [13], ensuring that the intervention is grounded in clearly identifiable active ingredients. These components are delivered within structured group workshops designed to elicit experiential reflection, promote volitional goal-setting, and cultivate a non-evaluative social context, factors shown to enhance need satisfaction and autonomous motivation in health contexts [14,15].

A distinctive contribution of this trial is the integration of functionality-oriented body image content. Functionality-focused interventions shift attention from appearance to bodily capabilities, fostering more adaptive self-perception and reducing risk factors associated with EDs [16–18]. This approach is theoretically synergistic with SDT because functionality appreciation promotes intrinsic values, self-compassion, and personal meaning—core drivers of autonomous motivation. Moreover, by reducing appearance-based exercise motives, the intervention may help prevent maladaptive patterns such as compulsive exercise or guilt-driven activity [8,9].

Methodologically, the study incorporates several features that enhance rigor, transparency, and reproducibility. The randomized controlled design with a control group aligns with SPIRIT 2025 guidelines, while analyst blinding reduces bias—a procedure rarely implemented in SDT-based trials [14,15]. The measurement battery includes validated Spanish instruments with strong psychometric properties—BPNSFS, BREQ-3, IPAQ-SF, BAS-2, and EAT‑26—ensuring reliable assessment of motivational, behavioral, and psychological constructs [26,28–30,32]. In particular, the use of the BPNSFS allows the examination of both need satisfaction and need frustration, enabling exploration of the “bright and dark paths” of psychological functioning within the SDT framework [28]. Intervention fidelity is supported through facilitator training, session guides, and structured checklists, enhancing the replicability of the program. Together, these procedures aim to strengthen internal validity while facilitating transparency and reproducibility of the intervention design.

Nevertheless, certain limitations should be acknowledged. Conducting the trial within a single university may restrict generalizability to other cultural or institutional contexts, particularly given known differences in PA norms and body image constructs across cultures [28,30]. Blinding of participants and facilitators is not feasible, common in educational and psychosocial interventions, which may introduce social desirability bias [6,15]. The use of self-report measures poses additional challenges: IPAQ-SF may overestimate vigorous PA [29], while the EAT‑26 identifies risk but does not provide diagnostic classification [32]. Finally, although the six-month follow-up assesses medium-term maintenance, longer-term evaluations would be necessary to examine deeper internalization processes and the stability of motivational regulation over time [16]. Because all eligible students are invited (census invitation), self-selection operates at the response rather than the invitation stage: who respond may already be more motivated, which threatens external validity (generalisability) more than the internal validity of the randomised comparison. Rather than merely acknowledging this, the response rate is reported, respondents are compared with the full eligible population, and a post-stratification / inverse-probability-of-response weighting sensitivity analysis probes the robustness of estimates to volunteer bias [37,38]. Residual selection on unobserved characteristics (e.g., baseline motivation) cannot be fully excluded.

Beyond these limitations, this protocol also contributes to ongoing theoretical discussions within SDT research. While the present study focuses on the roles of need satisfaction and need frustration, emerging conceptual work has proposed the additional construct of “need unfulfillment,” describing situations in which psychological needs are neglected rather than actively thwarted [39]. Future research could extend the present design to examine how these distinct need states influence motivational processes in physical activity contexts.

Despite these limitations, the present trial has the potential to make meaningful contributions. If effective, it may offer a scalable, theory-driven model for promoting sustainable PA engagement and positive body image in young women, a group affected by appearance pressures and ED risk [5,7].

Additionally, targeting Health Sciences students has strategic value: improving their autonomous regulation may foster autonomy-supportive communication styles in their future clinical practice, contributing to more person-centered healthcare [10,12]. The treatment of personal data will strictly comply with [33], ensuring the reconciliation of the right to data protection with the freedom of scientific research. Planned dissemination through peer-reviewed publications, conference presentations, and institutional reports will ensure that the findings contribute to both scientific knowledge and the development of theory-informed health promotion strategies in university and healthcare settings.

## Supporting information

S1 File SPIRIT 2025 Checklist. (DOCX)

S2 File TIDieR Checklist. (DOCX)

S3 File Statistical Analysis Plan (SAP), Version 2.0. (PDF)

S4 File Ethics Committee Report (English version). (DOCX)

S5 File Ethics Committee Report (Spanish version). (DOCX)
