# Supplementary material for: Study protocol for a randomized controlled trial of a self-determination theory–based psychoeducational intervention to enhance autonomous motivation for exercise in female university students
Source: PLoS One. 2026 Aug 7;21(8):e0355374. doi: 10.1371/journal.pone.0355374 (PMC13450776; doi:10.1371/journal.pone.0355374)
Supplement: Supplementary file 1 [file pone.0355374.s003.pdf]

Supplementary Material (S3 File)

Statistical Analysis Plan (SAP)

Study protocol for a randomized controlled trial of a self-determination theory–based psychoeducational intervention to enhance autonomous motivation for exercise in female university students *(NCT07506343)*

The following table presents the administrative details and the general framework of the institutional randomized controlled trial:

Table 1. General information and clinical trial registration.

Field	Detail
Study title	Study protocol for a randomized controlled trial of a self-determination theory–based psychoeducational intervention to enhance autonomous motivation for exercise in female university students
Trial registration	ClinicalTrials.gov NCT07506343
Ethics approval	Research Ethics Committee, Universidad Francisco de Vitoria (ref. 29/2026)
Funding	Universidad Francisco de Vitoria (UFV), competitive internal research call (ref. UFV2026-22).
Sponsor	Investigator-initiated; sponsored institutionally by Universidad Francisco de Vitoria. No external commercial sponsor. The funder (UFV) provided financial support through a competitive award and had no role in study design, data collection and analysis, decision to publish, or preparation of the manuscript.
Intervention	Six-session group psychoeducational program based on Self-Determination Theory with functionality-oriented body image content
SAP version / date	Version 2.0 — [13 July 2026]
Author(s)	S.G.M; P.R.B; G.D.U (Faculty of Health Sciences, UFV)

1. Introduction and Purpose

This Statistical Analysis Plan (SAP) pre-specifies, in full technical detail, the statistical methods for the randomized controlled trial of a Self-Determination Theory (SDT)–based psychoeducational intervention designed to enhance autonomous motivation for exercise and psychological well-being among female university students. It defines the analysis populations, endpoints, statistical models, handling of missing data, and sensitivity analyses, so that the planned analyses can be reproduced and any post-hoc analysis is clearly identified as such.

Any analysis not pre-specified here will be reported as post-hoc and exploratory; any deviation from these methods will be documented, justified, and reported in the final manuscript (Section 14).

2. List of Abbreviations

To facilitate the technical reading of this document, the acronyms used throughout the text are detailed along with their respective definitions in the following table:

Table 2. List of abbreviations and glossary of statistical and clinical terms.

Abbreviation	Definition
ANCOVA	Analysis of covariance
BAS-2	Body Appreciation Scale-2
BPNSFS	Basic Psychological Need Satisfaction and Frustration Scale
BREQ-3	Behavioral Regulation in Exercise Questionnaire-3
CACE	Complier Average Causal Effect
CI	Confidence interval
EAT-26	Eating Attitudes Test-26
GLMM	Generalized linear mixed model
ICC	Intraclass correlation coefficient
IPAQ-SF	International Physical Activity Questionnaire – Short Form
IPW	Inverse-probability (of response) weighting
ITT	Intention-to-treat
LSmean	Least-squares (estimated marginal) mean
MAR / MCAR / MNAR	Missing at random / completely at random / not at random
MET	Metabolic equivalent of task
MMRM	Mixed model for repeated measures
MSEM	Multilevel structural equation model
PA	Physical activity
PP	Per-protocol
RAI	Relative Autonomy Index
REML	Restricted maximum likelihood
SEM	Structural equation model
SDT	Self-Determination Theory
SD / SE	Standard deviation / standard error

3. Study Design and Objectives

This is a single-centre, two-arm, parallel-group randomized controlled trial with 1:1 allocation to (a) the experimental group, receiving a six-session SDT-based psychoeducational program with functionality-oriented body image content, or (b) a wait-list control group, which receives the full program after the final assessment. Outcomes are assessed at baseline (T0), immediate post-intervention (T1), and six-month follow-up (T2).

3.1 Objectives and Hypotheses

1. H1 (primary). The intervention increases autonomous motivation for PA (BREQ-3 RAI) at T1 and T2.
2. H2. It increases basic psychological need satisfaction (BPNSFS) at T1 and T2.
3. H3. It increases PA level (IPAQ-SF) at T1 and T2.
4. H4. It improves positive body image (BAS-2) at T1 and T2.
5. H5. It reduces eating disorder risk (EAT-26) at T1 and T2.
6. H6 (mechanism). Change in autonomous motivation mediates the intervention's effect on PA (IPAQ-SF) and eating disorder risk (EAT-26).

3.2 Primary Estimand (ICH E9 R1)

The primary research question is framed as an estimand to remove ambiguity about how intercurrent events are handled. In accordance with the ICH E9 (R1) guidelines, the methodological design is operationalized through the specific attributes of the primary estimand outlined in the following table:

Table 3. Specifications of the primary estimand attributes according to the ICH E9 (R1) framework.

Estimand attribute	Specification for the primary estimand
Treatment	SDT-based six-session program vs. wait-list control, as randomized (treatment-policy strategy)
Population	Female Health Sciences students aged 18–25 meeting eligibility (ITT population)
Variable (endpoint)	Relative Autonomy Index (RAI) from the BREQ-3 at T2 (six-month follow-up)
Intercurrent events	Non-adherence, use of external services (co-interventions), and withdrawal handled by the treatment-policy strategy; the MMRM uses all observed data under MAR
Population-level summary	Between-group difference in adjusted (least-squares) mean RAI at T2, with 95% CI

4. Endpoint

The study protocol outlines the evaluation of variables across multiple timepoints, hierarchically classified in the following table:

Table 4. Classification of study endpoints, outcome measures, and assessment timepoints.

Endpoint role	Domain	Measure (metric)	Timepoint(s)
Primary	Autonomous motivation for PA	BREQ-3 – RAI (between-group adjusted LSmean difference)	T2
Secondary – maintenance	Autonomous motivation for PA	BREQ-3 – RAI	T1
Secondary	Need satisfaction/frustration	BPNSFS subscales	T1, T2
Secondary	Physical activity	IPAQ-SF total MET-min/week (absolute)	T1, T2
Secondary	Positive body image	BAS-2 total	T1, T2
Secondary	Eating disorder risk	EAT-26 total score (continuous; ≥20 reported descriptively)	T1, T2
Exploratory	Motivational regulations	Six BREQ-3 subscales	T1, T2
Exploratory (mechanism)	Mediation	Indirect effect of group on PA (IPAQ-SF), ED risk (EAT-26), and body image (BAS-2) via change in RAI	T0→T1→T2

5. Sample Size Justification

5.1 Initial Approximation

An initial sample size was obtained with a repeated-measures ANOVA framework in G*Power (v3.1.9.6), assuming a small effect size ($f = 0.10$), two-sided $\alpha = 0.05$, power $(1 - \beta) = 0.80$, two groups, and three measurements (T0, T1, T2). This yielded a minimum of 164 participants (82 per group); inflating for ~20% attrition gave a recruitment target of 196 (≈98 per group).

5.2 Confirmation Under the Primary Model (MMRM)

Repeated outcomes at T0, T1 and T2 were drawn with a between-time correlation ρ; variance was decomposed into a between-subject component ($SD = \sqrt{\rho}$) and a within-subject residual ($SD = \sqrt{(1 - \rho)}$), so the marginal SD = 1. The experimental arm received the effect at T1 and a partial maintenance effect at T2. Monotone, missing-at-random dropout of 10% at T1 and a further 10% by T2 was imposed. Each data set was analysed with the planned MMRM (fixed effects: group, time, group × time, baseline as covariate; unstructured covariance; REML), and power was the proportion of replicates in which the between-group LSmean contrast at T2 reached $p < 0.05$. Two thousand replicates were run per scenario with a fixed seed, using the mmrm and emmeans R packages.

5.3 Assumed Parameters and Their Justification

The primary scenario assumed d = 0.35 at T2, ρ = 0.70 and 10% attrition at T1. The planning effect is anchored on empirical evidence: meta-analytic estimates for SDT-based interventions on motivation are g ≈ 0.41, and for body-image interventions g ≈ 0.40, so d = 0.35 is conservative. The correlation ρ = 0.70 is consistent with the temporal stability reported for the BREQ-3 in Spanish samples.

5.4 Results (Primary Scenario: d = 0.35, ρ = 0.70)

Simulation results for the planned primary scenario ($\boldsymbol{d = 0.35}$; $\boldsymbol{\rho = 0.70}$) estimate the relationship between sample size and statistical power as follows:

Table 5. Statistical power calculations by sample size for the primary planned scenario.

n	Total n	Power (T2 contrast)
60	120	0.60
70	140	0.68
80	160	0.74
90	180	0.79
98	196 (target)	≈0.82
100	200	0.82
110	220	0.86
120	240	0.89

The planned sample (196; ≈98 per group) provides approximately 0.82 power for the primary between-group contrast at T2; the 0.80 threshold is reached at ≈93 per group.

5.5 Sensitivity Analysis

Under a more conservative effect (d = 0.30), power is approximately 0.72–0.73 (ρ = 0.60–0.70); under d = 0.35 it is approximately 0.82 at both ρ = 0.60 and ρ = 0.70. The trial is therefore adequately powered (≥0.80) for effect sizes most consistent with prior SDT and body-image interventions.

Consequently, while the trial targets a conservative scenario ($d = 0.35$), a minimum sample size of 70 participants per group has been established as the absolute lower bound for acceptable recruitment. Based on the meta-analytic literature for identical interventions ($g \approx 0.40$), supplementary simulations confirm that a sample of $N = 70$ per group still provides adequate statistical power ($1 - \beta \geq 0.80$) to detect this expected true effect size.

Table 6. Power sensitivity analysis and realized effect sizes for the minimum sample size lower bound.

n	Total n	Power	Realized *g*
40	80	0.54	0.399
50	100	0.67	0.405
60	120	0.72	0.398
70	140	0.80	0.404
80	160	0.86	0.403
90	180	0.88	0.396

5.6 Precision, Reproducibility, and Clustering

With ≈98 per group (≈88 completers at T1), the 95% CI for the standardized between-group difference has a half-width of approximately ±0.20 SD. The simulation script and random seed are archived with the trial materials. Because the experimental program is delivered in fixed subgroups (Section 8.1), the design is partially nested; clustering may modestly reduce the effective sample size, so the realized power may be slightly lower than the value above. This is examined in the clustering sensitivity analysis (Section 9.2) and acknowledged as a limitation.

6. Randomization, Blinding, and Allocation

Following baseline assessment (T0), participants are allocated 1:1 to the experimental or control group. The allocation sequence is generated with a computerized random-number generator in R (blockrand package), using a fixed seed, by an independent researcher not involved in recruitment, delivery, or outcome assessment. Randomization uses permuted blocks of variable, concealed size (4, 8, and 12; 1:1 within each block), without stratification. Allocation concealment is ensured by storing the sequence in a secure, access-restricted file revealed only after T0 and eligibility confirmation.

Blinding by role. Participants and providers (facilitators) cannot be blinded given the nature of a group psychoeducational program. Outcomes are self-reported with standardized instruments (no clinician-rated outcomes). The data manager and statistician remain blinded to allocation via coded group labels until the primary analysis is finalized; the blind is broken only after the analysis program is locked.

Recruiting and enrolling staff remain masked to the allocation sequence and to the block sizes throughout the trial. As a post-hoc verification of the absence of allocation (third-order) selection bias, a Berger–Exner test will be applied to check for any association between the allocation sequence and baseline prognostic characteristics.

7. Analysis Populations

1. Intention-to-treat (ITT) – primary. All randomized participants with at least one post-baseline measurement, analysed as randomized. The MMRM contributes all observed data.
2. Per-protocol (PP) – sensitivity. Participants attending all six sessions (missing any session is non-adherence) with no major protocol deviation.

The complier-average-causal-effect (CACE) is estimated as a sensitivity analysis (Section 9.2) in the latent subgroup of compliers, using randomization as an instrument. Participant flow is reported in a CONSORT diagram. Given the low-risk behavioral intervention, no separate safety analysis set is required.

8. Statistical Analyses

8.1 General Principles

All analyses are pre-specified prior to database lock and conducted in RStudio (v.2026.01.1+403, Posit Software). Planned packages are mmrm and emmeans (continuous MMRM and least-squares means), glmmTMB (generalized linear mixed models, including Tweedie and hurdle/zero-inflated families), lavaan, semTools and mediation (mediation and Monte Carlo confidence intervals), and performance and DHARMa (assumption checks); lme4 and nlme are optional. No multiple-imputation package is required, because the primary analysis does not impute and the missing-data sensitivity (Section 9.2) is implemented without imputation. Continuous variables are summarized as n, mean, SD, median, IQR, minimum and maximum; categorical variables as counts and percentages. Two-sided p-values and 95% CIs are reported; significance for the primary hypothesis is $\alpha = 0.05$.

Partially nested structure. The trial has a partially nested structure: experimental-arm participants are delivered the program in fixed subgroups (the same composition is retained across the six sessions), whereas control participants are not. This clustering is kept out of the primary models and addressed through pre-specified sensitivity analyses — a delivery-group random intercept for the continuous outcomes (Section 9.2) and a multilevel SEM for the mediation (Section 8.4). The clustering is relevant to any analysis using the experimental arm (primary, secondary, and mediation) and is handled consistently as a sensitivity analysis in all of them.

8.2 Primary Efficacy Analysis – MMRM

The primary endpoint (RAI at T2) is analysed with an MMRM including all post-randomization assessments of the primary outcome:

- Fixed effects: group (experimental vs. control), time (T1, T2) as a categorical factor, group × time, and baseline (T0) RAI as a covariate.

- Covariance: unstructured; if it fails to converge, pre-specified fallbacks (heterogeneous Toeplitz, then AR(1)) are tried, selecting the convergent model with lowest AICc.
- Estimation: REML, with Satterthwaite degrees-of-freedom adjustment for the fixed-effects tests.
- Treatment effect: least-squares mean difference between groups at T2 (primary) and T1 (intermediate secondary), via emmeans, with 95% CIs and p-values.

The intervention effect is estimated by the group × time interaction — equivalently, the between-group least-squares-mean difference at each post-baseline timepoint. Because baseline (T0) RAI is measured prior to randomization, including it as a covariate increases precision and corrects for any chance baseline imbalance without absorbing the treatment effect.

This likelihood-based approach uses all observed data without imputing missing outcomes and yields valid inference under MAR.

8.3 Secondary Analyses

Each secondary continuous outcome (BPNSFS, BAS-2, EAT-26 total, IPAQ-SF) uses the same MMRM structure, producing adjusted between-group differences at T1 and T2. The analytic form respects the distribution of each instrument: (1) default continuous MMRM on the raw score if residual diagnostics are acceptable; (2) a normalizing transformation (log or square-root) if moderately skewed; (3) IPAQ-SF absolute MET-min/week analysed with a GLMM (glmmTMB) using a Tweedie or hurdle/zero-inflated distribution, which accommodates continuous, non-negative, right-skewed values with exact zeros; (4) EAT-26 continuous total analysed with the MMRM, with a normalizing transformation if residuals are skewed, and the ≥20 risk threshold reported descriptively. Where assumptions remain violated after transformation, robust mixed-effects estimation and/or non-parametric bootstrap (5,000 resamples) are used. The six BREQ-3 subscales are analysed exploratorily with the same approach.

8.4 Mediation Analysis (Mechanism, H6)

H6 is tested with a half-longitudinal mediation model. Group assignment is the independent variable (X); the change in autonomous motivation (BREQ-3 RAI) from T0 to T1 is the mediator (M); and the changes in physical activity (IPAQ-SF), eating disorder risk (EAT-26), and positive body image (BAS-2) from T1 to T2 are the outcomes (Y), each modelled separately. The mediator is measured before the outcome, supporting temporal precedence.

Primary specification. The model is estimated as a single-level structural equation model in lavaan, with two linked equations — a mediator model (M on X) and an outcome model (Y on X and M) — both adjusting for the corresponding baseline value, using a robust (MLR) estimator. The indirect effect a×b is estimated with bias-corrected and accelerated bootstrap confidence intervals (5,000 resamples); the total effect and the proportion mediated are reported.

Sensitivity to clustering (MSEM). Because the experimental program is delivered in subgroups, experimental-arm participants are nested within delivery groups whereas wait-list control participants are not (a partially nested structure). As a sensitivity analysis, the mediation is re-estimated as a multilevel structural equation model (MSEM) with a random intercept for delivery group in the experimental arm, the indirect effect obtained with Monte Carlo confidence intervals; agreement with the primary single-level estimate is reported.

Causal assumptions. Because only group is randomized (not the mediator), the mediator→outcome path is observational; the analysis is exploratory and rests on sequential-ignorability assumptions (no unmeasured mediator–outcome confounding), for which a sensitivity analysis quantifying robustness to unmeasured confounding is reported (mediation package). As a further exploratory extension, the upstream SDT pathway (need satisfaction → autonomous motivation) may be examined as a serial mediation, acknowledging that the two mediators are measured concurrently and therefore provide weaker temporal separation.

9. Missing Data and Sensitivity Analyses

9.1 Missing Data Strategy

The extent and pattern of missing data will be described by group and time, and baseline characteristics of completers vs. non-completers compared to inform the missingness mechanism.

9.2 Pre-specified Sensitivity Analyses

To ensure the robustness of the findings against potential violations of assumptions, the pre-specified analytical strategies are outlined in the following table:

Table 7. Pre-specified sensitivity analyses and missing data handling strategies.

Sensitivity analysis	Purpose / concern	Method
Per-protocol (PP)	Non-adherence / fidelity	Repeat primary MMRM in participants attending all six sessions
Adherence as continuous	Dose–response (exploratory)	MMRM adding attendance proportion and its interaction with group (non-causal)
Fidelity-adjusted	Delivery fidelity	MMRM including a session fidelity score as covariate
CACE / complier	Wait-list dissatisfaction & non-adherence	Instrumental-variable / latent-class CACE using randomization as instrument
Co-intervention adjustment	Contamination from external services	MMRM adding self-reported external-service use as a time-varying covariate
Alternative covariance	Robustness of within-subject model	Refit with compound symmetry and AR(1); compare estimates
Model specification	Robustness to adjustment choices	Refit without baseline adjustment and with baseline × treatment interaction
Clustering (partially nested)	Group-delivered intervention in the experimental arm	Refit the primary and key secondary MMRMs adding a random intercept for delivery group in the experimental arm (control participants as size-1 clusters); report the delivery-group ICC and compare with the unclustered models
Representativeness / volunteer bias	Voluntary response within a census-invited frame	Compare respondents with the full invited eligible population on available administrative variables (degree program, year of study); post-stratify / rake estimates to the population margins; report overall and stratum-specific response rate
Missing-data (MNAR)	Departure from MAR	Delta-adjusted / pattern-mixture tipping-point applied analytically to the MMRM means (no imputation)

9.3 Wait-list Bias and Potential Overestimation

Several features bound and probe this risk: (i) the primary estimand is the ITT, treatment-policy effect, generally conservative for efficacy and preserving randomization; (ii) the MMRM adjusts for the baseline outcome, reducing the influence of any baseline imbalance from self-selection; (iii) the CACE/instrumental-variable analysis estimates the effect among compliers without breaking randomization; (iv) self-reported external-service use is entered as a time-varying covariate; and (v) divergence between ITT, PP, and CACE estimates is reported as an explicit indicator of possible over- or under-estimation.

Because all eligible students are invited (census invitation), self-selection operates at the response rather than the invitation stage. Representativeness is therefore evaluated by comparing respondents with the full invited eligible population and by a post-stratification/inverse-probability-of-response weighting sensitivity analysis (Section 9.2). This corrects bias on observed variables only; residual selection on unobserved characteristics (e.g., baseline motivation) cannot be fully excluded. Interpretation will state plainly that wait-list designs can inflate apparent effects.

9.4 Co-interventions / Concomitant Care

Use of external services is collected at each assessment, summarized by arm, and tested for between-arm imbalance; if imbalance or association with outcomes is observed, the co-intervention-adjusted MMRM is reported alongside the primary result.

10. Derived Variables and Scoring Conventions

BREQ-3 RAI is the weighted sum of the six subscale means using standard weights (amotivation −3, external −2, introjected −1, identified +1, integrated +2, intrinsic +3; range −24 to +24). BPNSFS satisfaction and frustration subscale scores are item means. IPAQ-SF total MET-min/week uses the standard algorithm (walking 3.3, moderate 4.0, vigorous 8.0 MET) with daily minutes truncated at 180; activity is also classified as low/moderate/high. BAS-2 and EAT-26 totals are item sums; EAT-26 risk is a total ≥ 20. Item-level missingness within an instrument is handled by that instrument's validated rule (pro-rated mean where permitted); otherwise the scale score is set to missing for that assessment.

11. Reporting of Descriptive Statistics for Meta-analysis

Descriptive statistics, including sample size (n), mean, and standard deviation, will be reported for each group at each time point for all questionnaires and their corresponding subscale at baseline (T0), post-intervention (T1), and follow-up (T2)

12. Multiplicity and Type I Error Control

A single primary hypothesis (H1: RAI at T2) is tested at two-sided $\alpha = 0.05$; no adjustment is required. Secondary outcomes (H2–H5) and all T2 contrasts are pre-specified as exploratory and reported with unadjusted 95% CIs and p-values, with their exploratory status stated explicitly. No formal familywise adjustment is applied; the number and exploratory nature of comparisons are disclosed.

13. Statistical Software

All analyses use RStudio (v.2026.01.1+403, Posit Software) with the packages listed in Section 8.1 (including semTools for Monte Carlo confidence intervals). Analysis scripts and the power-simulation code (with fixed seed) are archived with the trial materials to support reproducibility.

14. Changes from the Protocol and Deviations

This SAP elaborates, and is consistent with, the protocol's statistical section, adding technical detail rather than changing the planned approach (covariance-structure selection, GLMM rules for non-normal secondary outcomes, CACE and co-intervention sensitivity analyses, half-longitudinal mediation with an MSEM clustering sensitivity, partially nested and representativeness sensitivity analyses, simulation-based power). Where the SAP provides more specific methods, this SAP takes precedence. Any subsequent deviation will be documented in the final report with its rationale, and substantive changes submitted to the UFV Research Ethics Committee and the trial registry.

15. SAP Roles, Version History, and Approval

Finally, the allocation of responsibilities among the authors for drafting, methodological review, and approval of this analysis plan is detailed in the following table:

Table 8. Statistical analysis plan roles, version history, and approvals.

Section / Attribute	Detail / Content
**1. Roles and Responsibilities**	
SAP primary author	GDU
Senior statistician (accountable)	GDU
Contributor(s)	SGM / co-author, PRB / co-author
**2. Version History**	
Version 1.0 (25 June 2026)	Pre-specified statistical analysis plan prior to database lock.
**3. Sign-off and Approvals**	
Technical approval and sign-off	Signed and approved by GDU
