# Supplementary material for: Study protocol for a randomized controlled trial of a self-determination theory–based psychoeducational intervention to enhance autonomous motivation for exercise in female university students
Source: PLoS One. 2026 Aug 7;21(8):e0355374. doi: 10.1371/journal.pone.0355374 (PMC13450776; doi:10.1371/journal.pone.0355374)
Supplement: Supplementary file 2 [file pone.0355374.s007.pdf]

Response to Reviewers

Manuscript: A randomized controlled trial of a Self-Determination Theory–based psychoeducational intervention to enhance autonomous motivation for exercise in female university students

We thank the Editor and both Reviewers for their careful and constructive evaluation of our manuscript. We have revised the protocol accordingly and have also prepared a separate Statistical Analysis Plan (SAP v1.1, Supplementary File S3), following the template proposed by Stevens et al. (2023). Below we respond point-by-point to all comments. References to "SAP" denote sections of the Statistical Analysis Plan. Page and line numbers refer to the revised manuscript with tracked changes visible.

Reviewer 1

We appreciate the reviewer's positive evaluation of the study rationale, design, sample size justification, data management procedures, and planned analyses.

Comment 1 — Retention procedures

> *"…although missing data is discussed there should also be a few words discussing what retention procedures will be used to minimize the amount of missing data during the study."*

Response. Retention procedures have now been explicitly described. The protocol now states that periodic email reminders, advance notification of sessions and assessment points, systematic attendance monitoring, and reminders regarding the importance of continued participation will be implemented to minimise attrition and missing data. Additional details are provided in the SAP retention section.

Changes made: Manuscript page 7, lines 152–159; SAP 9.1.

Comment 2 — Mediation analysis too vague

> *"…the discussion of the mediation analysis is a bit too vague and should be detailed further. What models specifically will apply in this context?"*

Response. The mediation analysis has been fully specified. The manuscript now states that mediation will be examined using a half-longitudinal model, with group allocation as the independent variable, T0–T1 change in autonomous motivation (BREQ-3 Relative Autonomy Index) as the mediator, and T1–T2 changes in physical activity (IPAQ-SF), positive body image (BAS-2), and eating disorder risk (EAT-26) as outcomes. The primary specification is a single-level structural equation model (SEM) implemented in lavaan, with bias-corrected bootstrap confidence intervals used to estimate indirect effects. In addition, a multilevel SEM accounting for partially nested intervention delivery subgroups has been pre-specified as a sensitivity analysis. Full methodological details are provided in the Statistical Analysis Plan (Supporting Information S3).

Changes made: Manuscript page 21, lines 464–471; SAP 8.4.

Reviewer 2

We thank Reviewer 2 (Dr. Ethan Sahker) for the detailed methodological and statistical recommendations, which have helped to strengthen both the protocol and the Statistical Analysis Plan.

Comment 1 — Self-selection, wait-list overestimation, and compensation

> *"…If all participants self-select into a trial about motivation, this seems to be contributing problematic bias… waitlist controls are already known to overestimate trial effects… Please rethink this and how to manage such biases and potential overestimation…"*

Response. The protocol now explicitly states that participants will not receive financial compensation. Participants allocated to the wait-list condition will receive delayed access to the complete intervention following completion of the follow-up assessment.

To address potential volunteer self-selection bias, the revised protocol clarifies that recruitment follows a census invitation approach in which all eligible students are contacted. Response rates will be reported, respondents will be compared against the eligible invited population using available administrative characteristics, and post-stratification/inverse-probability-of-response weighting sensitivity analyses will be conducted. The SAP additionally specifies ITT, per-protocol, and CACE analyses to evaluate the potential influence of self-selection and treatment adherence. The discussion section has also been expanded to explicitly acknowledge the possibility of effect overestimation associated with wait-list control designs.

Changes made: Manuscript page 7, lines 163–167; page 28, lines 616–623; SAP 9.2–9.3

Comment 2 — Provide a separate SAP (Stevens et al. template)

> *"…I recommend the authors create a separate Statistical Analysis Plan (SAP) and include this as a supplement. Stevens et al. (2023) provides a very nice SAP template…"*

Response. A dedicated Statistical Analysis Plan (SAP v1.1) has been developed and included as Supplementary File S3. The SAP follows the template proposed by Stevens et al. (2023) and provides detailed specifications regarding estimands, outcomes, primary and secondary analyses, handling of missing data, mediation analyses, sensitivity analyses, and reporting procedures.

Changes made: Manuscript page 21, lines 477–478; Supplementary File S3 (SAP).

Comment 3 — Single primary endpoint, secondary timepoints, and a means/SD table

> *"…choosing a single endpoint for the primary analysis… note all timepoints in secondary analyses… include a table with all measurement means and SDs for all timepoints…"*

Response. A single primary endpoint has now been defined. The primary analysis focuses on autonomous motivation (BREQ-3 Relative Autonomy Index) at post-intervention (T1). Analyses at T2 are explicitly defined as secondary analyses evaluating maintenance of intervention effects.

A shell table reporting means and standard deviations for all outcomes and timepoints has also been incorporated into the SAP.

Changes made: Manuscript page 21, lines 464–467; SAP 4, 11 and 12.

Comment 4 — Primary outcome by domain, measure, metric, and timepoint

> *"State the primary outcome in terms of domain, measure, metric, and timepoint…"*

Response. The primary outcome is now explicitly defined as follows: domain = autonomous motivation toward physical activity; measure = BREQ-3 Relative Autonomy Index (RAI); metric = between-group adjusted least-squares mean difference; primary timepoint = post-intervention (T1). (administration procude).

Changes made: SAP 3.2 and 4.

Comment 5 — MMRM instead of multiple imputation; GLMM for count/categorical

> *"MI is no longer the preferred method… MMRM is preferred for continuous outcomes, and GLMM for count/categorical… MI is unnecessary if using MMRM."*

Response. The manuscript and SAP have been revised accordingly. Multiple imputation has been removed as the primary strategy for handling missing data. The primary analysis is now specified as a Mixed Model for Repeated Measures (MMRM) under a Missing At Random (MAR) assumption, incorporating all observed data without imputation. For outcomes that do not satisfy distributional assumptions, appropriate generalized mixed models are specified.

Changes made: Manuscript page 19, lines 410–416; SAP 8.2, 8.3 and 9.1.

Comment 6 — Allocation concealment

> *"…This statement does not describe allocation concealment. Please describe how the allocation sequence was concealed until participants were enrolled and assigned…"*

Response. Allocation concealment procedures have now been described in detail. The allocation sequence will be generated by an independent researcher not involved in recruitment, intervention delivery, or outcome assessment. The sequence will remain inaccessible to recruitment staff and will be revealed only after confirmation of eligibility and completion of baseline assessment. The protocol further specifies the use of variable block sizes and includes a pre-specified Berger–Exner assessment of allocation-selection bias.

Changes made: Manuscript page 10, lines 233–257; SAP 6.

Comment 7 — Consolidate Randomization, Blinding, and Allocation; blinding by level

> *"…make a subheading, 'Randomization, Blinding, and Allocation,' and mention everything once… mention how blinding is conducted at: Provider, participant, outcome assessor, researcher, data analyst levels."*

Response. Information regarding randomization, allocation concealment, and blinding has been consolidated into a single subsection entitled Randomization, Blinding, and Allocation. Blinding

procedures are now explicitly described for participants, intervention providers, researchers, and data analysts.

Changes made: Manuscript page 10, lines 233–257; SAP 6.

Comment 8 — Subheading for the Wait-list Control

> *"Create a subheading for the Wait-list Control."*

Response. The intervention section has been reorganized to improve clarity and readability. In addition to incorporating a dedicated subheading for the Wait-list control condition, a corresponding Experimental intervention subheading has also been introduced. This structure allows both study conditions to be presented consistently and facilitates identification of the intervention and comparator arms throughout the protocol.

Changes made: Experimental intervention: line 270; Wait-list control condition: line 333.

Comment 9 — Sensitivity analysis for fidelity

> *"I recommend a sensitivity analysis that accounts for fidelity issues if present."*

Response. Fidelity-related sensitivity analyses have been pre-specified. In addition to the primary ITT analysis, fidelity-adjusted, adherence-based, and per-protocol analyses will be conducted to evaluate the robustness of findings in relation to intervention delivery and participant attendance. Further details are provided in the SAP.

Changes made: Manuscript page 20, lines 428–430; SAP 9.2.

Comment 10 — Redundancy in study timepoints

> *"There is also redundancy with noting of the study timepoints that can be streamlined."*

Response. Descriptions of T0, T1 and T2 have been reviewed throughout the manuscript and streamlined where possible to reduce redundancy while maintaining consistency across sections.

Changes made: Revisions throughout the manuscript.

Comment 11 — Deviations and concomitant/contaminant care

> *"…they do not mention what they will do about contaminant care or what is allowed… Are these data collected and perhaps accounted for in sensitivity analyses…?"*

Response. The protocol now explicitly states that participants may continue accessing external services, including psychological support, personal training, dietary counselling, and other health-related services. These data will be collected at each assessment, summarised by treatment arm, evaluated for imbalance, and incorporated into sensitivity analyses as time-varying covariates where appropriate. Protocol deviations and co-interventions will be documented and reported.

Changes made: Manuscript page 16, lines 337–346; SAP 9.2 and 9.4.

Comment 12 — SPIRIT checklist N/A items and trial sponsor

> *"…address each of these in the Protocol. If they are truly N/A, state why… For example, 'Name and contact information for the trial sponsor.' If this was an unfunded study, state this."*

Response. The sponsor and funding source have now been explicitly reported in the protocol (Universidad Francisco de Vitoria; internal competitive funding call UFV2026-22). In addition, all SPIRIT checklist items previously marked as "N/A" were reviewed individually.

Several items that were previously marked as "N/A" have been completed following the reviewer's recommendation. For those items that remain designated as "N/A", we consider this designation appropriate given the characteristics of the present trial. Specifically, item 3d was retained as N/A because no steering committee, endpoint adjudication committee, or data monitoring committee has been established, as this is a single-centre, low-risk behavioural intervention coordinated directly by the principal investigators. Item 11 was retained as N/A because patients or members of the public were not involved in the design, conduct, reporting, or dissemination plans of this study, which was developed from previous empirical evidence and behavioural theory. Given the single-centre design, the behavioural nature of the intervention, and the minimal risks involved, oversight by an independent DMC is not considered necessary. Item 28b remains N/A because no interim analyses or stopping guidelines are planned owing to the low-risk nature and relatively short duration of the intervention. Item 32b also remains N/A because no biological specimens will be collected and no ancillary studies are planned; therefore, no additional consent beyond participation in the main trial is required.

Changes made: Funding section (manuscript page 1); revised SPIRIT checklist (S1 items 3d, 11, 28b, and 32b); SAP header
