# Supplementary material for: Study protocol for a randomized controlled trial of a self-determination theory–based psychoeducational intervention to enhance autonomous motivation for exercise in female university students
Source: PLoS One. 2026 Aug 7;21(8):e0355374. doi: 10.1371/journal.pone.0355374 (PMC13450776; doi:10.1371/journal.pone.0355374)
Supplement: Supplementary file 4 [file pone.0355374.s002.docx]

The TIDieR (Template for Intervention Description and Replication) Checklist*:

Information to include when describing an intervention and the location of the information

m

|  |  |  | Where located ** |
| --- | --- | --- | --- |
| Ítem number | Ítem | Description | Primary paper (page or appendix number) |
| 1. | **BRIEF NAME:** Provide the name or a phrase that describes the intervention. | SDT-Based Intervention for Exercise Motivation in Female Students | 8-13 |
| 2. | **WHY**: Describe any rationale, theory, or goal of the elements essential to the intervention. | The intervention is grounded in Self-Determination Theory and functionality-based body image approaches, aiming to enhance autonomous motivation, psychological need satisfaction, and well-being while reducing maladaptive exercise motives and body-related concerns. | 3-5, 8-9 |
| 3. | **WHAT**: **Materials**: Describe any physical or informational materials used in the intervention, including those provided to participants or used in intervention delivery or in training of intervention providers. Provide information on where the materials can be accessed (e.g. online appendix, URL). | The intervention includes structured presentation slides, participant worksheets, guided experiential activities, and reflective exercises. Materials are delivered during sessions and are available upon request; detailed session content is summarized in Tables 1 and 2. | 10-13 |
| 4. | **WHAT**: **Procedures**: Describe each of the procedures, activities, and/or processes used in the intervention, including any enabling or support activities. | The intervention consists of six structured face-to-face group sessions combining psychoeducational content, guided reflection, experiential tasks, and group discussion. Each session follows a predefined structure aligned with SDT principles and body functionality content. | 11-13 |
| 5. | **WHO** **PROVIDED**: For each category of intervention provider (e.g. psychologist, nursing assistant), describe their expertise, background and any specific training given. | The intervention is delivered by trained facilitators with expertise in exercise science and psychonutrition, who received received prior structured training in intervention delivery and SDT-based communication strategies. | 9 |
| 6. | **HOW**: Describe the modes of delivery (e.g. face-to-face or by some other mechanism, such as internet or telephone) of the intervention and whether it was provided individually or in a group. | The intervention is delivered face-to-face in small group workshop format, promoting interaction, reflection, and peer discussion. | 9 |
| 7. | **WHERE**: Describe the type(s) of location(s) where the intervention occurred, including any necessary infrastructure or relevant features. | The intervention takes place at Universidad Francisco de Vitoria (Madrid, Spain), in classroom settings suitable for group-based sessions. | 6 |
| 8. | **WHEN** and **HOW** **MUCH**: Describe the number of times the intervention was delivered and over what period of time including the number of sessions, their schedule, and their duration, intensity or dose. | The intervention comprises six sessions of approximately 60 minutes each, delivered over a defined intervention period. | 9 |
| 9. | **TAILORING**: If the intervention was planned to be personalised, titrated or adapted, then describe what, why, when, and how. | The intervention allows minor adaptations in delivery (e.g., examples or discussion prompts) to fit group dynamics, while maintaining core theoretical components and session structure. | 8-9 |
| 10. | **MODIFICATIONS**: If the intervention was modified during the course of the study, describe the changes (what, why, when, and how). | Not applicable, as this is a study protocol and no modifications have yet been made. | N/A |
| 11. | **HOW** **WELL**: **Planned**: If intervention adherence or fidelity was assessed, describe how and by whom, and if any strategies were used to maintain or improve fidelity, describe them. | Intervention fidelity will be monitored using standardized session checklists completed by facilitators, ensuring adherence to core components and delivery principles. | 11 |
| 12. | **HOW** **WELL**: **Actual**: If intervention adherence or fidelity was assessed, describe the extent to which the intervention was delivered as planned. | Not applicable at the protocol stage; fidelity outcomes will be reported in future trial results. | N/A |

** **Authors** - use N/A if an item is not applicable for the intervention being described.
