# Supplementary material for: Study protocol for a randomized controlled trial of a self-determination theory–based psychoeducational intervention to enhance autonomous motivation for exercise in female university students
Source: PLoS One. 2026 Aug 7;21(8):e0355374. doi: 10.1371/journal.pone.0355374 (PMC13450776; doi:10.1371/journal.pone.0355374)
Supplement: Supplementary file 5 [file pone.0355374.s004.docx]

**PROJECT REPORT FOR THE ETHICS COMMITTEE**

**Study title:**
Effect of a Psychoeducational Intervention Based on Self-Determination Theory on Motivation for Physical Exercise and Healthy Eating in Female Health Sciences University Students.

**Principal Investigator:** Dr. Sonia García Merino
**Co-Director:** Germán Díaz Ureña

**Center / Faculty:** Faculty of Health Sciences

**Email:** sonia.garciamerino@ufv.es

**Planned duration:** January 2026 – December 2026

**1. INTRODUCTION: JUSTIFICATION AND CONTEXT OF THE STUDY**

The first years of university represent a transitional stage that can significantly alter young people’s eating and physical activity habits. Several longitudinal studies have shown that this period is associated with a decline in physical activity and increased irregularity in dietary patterns (Deforche et al., 2015; Finlayson et al., 2012). In this context, the motivations underlying health-related behaviors become particularly relevant among university populations. It is common for exercise practice and dietary control to be driven by aesthetic or social purposes rather than by well-being or self-care motives (Diehl et al., 2018). Understanding the types of motivation that sustain these behaviors is therefore essential, as the way individuals relate to exercise and nutrition directly influences the quality of their habits and their psychological well-being.

From this perspective, Self-Determination Theory (SDT; Deci & Ryan, 2000) provides a robust explanatory framework for analyzing different types of motivation toward health-related behaviors. According to this theory, the quality of motivation—autonomous versus controlled—determines adherence and the psychological impact of such behaviors. When individuals are motivated by personal interest and choice (autonomous motivation), they tend to maintain behaviors more sustainably and experience greater emotional well-being. Conversely, controlled motivations (e.g., guilt, pressure, or desire for approval) are associated with lower satisfaction and an increased risk of maladaptive behaviors. Brief SDT-based interventions have been shown to enhance autonomous motivation and healthy habits among university students (Teixeira et al., 2020; Weman-Josefsson et al., 2015).

This study is embedded within the research line developed by our research group, whose previous results have advanced understanding of motivational factors linked to body image and health behaviors in university populations. In a first study published in Frontiers in Psychology (2025), sex differences were observed in body perception and eating disorder (ED) risk. Subsequently, a second study published in Healthcare (2025), focused on female university students, revealed a positive association between physical activity level and EAT-26 scores, although values did not reach pathological thresholds. These findings suggest that part of physical exercise practice may be guided by controlled motives, such as aesthetic concerns or social pressure, rather than by autonomous motives related to health and well-being. Based on these results, a third study is proposed to evaluate the effect of a psychoeducational intervention based on Self-Determination Theory, aimed at promoting more autonomous motivation toward exercise and healthy eating, fostering healthy habits and preventing risk behaviors related to body image.

**2. OBJECTIVES**

**General objective:**

To evaluate the effect of a group-based psychoeducational intervention grounded in Self-Determination Theory on motivation for physical exercise and healthy eating in female Health Sciences university students.

**Specific objectives:**

- To analyze changes in motivation toward exercise (BREQ-3; RAI) and eating behavior (REBS; RAI) following the intervention.
- To examine the effect on satisfaction and frustration of basic psychological needs (BPNSFS).
- To assess the impact on physical activity level (IPAQ-short), eating disorder risk (EAT-26), and body dissatisfaction/body appreciation (BAS-2; Body Appreciation Scale-2).
- To determine whether motivational changes mediate the relationship between the intervention and improvements in exercise and eating habits.

**3. METHODOLOGY**

3.1. DESIGN

A randomized controlled trial will be conducted with three measurement points: pre-intervention (T0), post-intervention (T1), and six-month follow-up (T2). Following baseline assessment, participants will be randomly allocated using simple random sampling via a random number generator implemented in RStudio (Version 2025.09.1+401, Posit Software). Participants will be assigned in a 1:1 ratio to either an intervention group or a waiting-list control group.

The intervention will consist of six face-to-face group workshops, each lasting 60 minutes, conducted between March and April 2026. The sessions will focus on healthy exercise, healthy eating, and autonomous motivation. The control group will not receive any intervention during the study period but will be offered the full program after completion of the follow-up assessment (T2).

3.2. PARTICIPANTS AND SELECTION CRITERIA

The target population will consist of students enrolled in the following undergraduate degrees at the Francisco de Vitoria University: Physical Activity and Sport Sciences, Physiotherapy, Human Nutrition and Dietetics, and Nursing.

The sample will include women aged between 18 and 25 years who are enrolled in any of the aforementioned degrees and who voluntarily agree to participate by signing the informed consent form.

Exclusion criteria will include failure to complete questionnaires at any measurement point (T0 or T1), attendance at less than 75% of the intervention sessions, and a current or previous diagnosis of eating disorders (ED or OSFED).

3.3. SAMPLE SIZE

Sample size was calculated a priori using G*Power software (version 2.9, University of Düsseldorf) with an F test design: ANOVA repeated measures, within-between interaction.

The following parameters were considered: small expected effect size (η²p = 0.01), significance level (α = 0.05), statistical power (1–β = 0.80), two comparison groups, three repeated measurements per participant, correlation among repeated measures (r = 0.50), and a non-sphericity correction of 1.

Based on these parameters, the required sample size was 162 participants (81 per group). To account for an anticipated attrition rate of 20%, the target sample size was set at 194 participants.

3.4. PROCEDURES AND MEASURES

The following questionnaires and scales will be administered:

- BREQ-3 (González-Cutre et al., 2010)
- REBS (Pelletier et al., 2004)
- BPNSFS (Cardella et al., 2020)
- IPAQ-short (Rodríguez-Muñoz et al., 2017)
- EAT-26 (Rivas et al., 2010)
- BAS-2 – Body Appreciation Scale-2 (Jáuregui Lobera et al., 2011)

The questionnaires will be administered in a sequence designed to minimize bias and protect participants’ well-being: first, the BPNSFS, given its general and emotionally neutral content; followed by the BREQ-3 and REBS to assess motivation toward exercise and eating; next, the IPAQ-short as an intermediate behavioral measure; then the BAS-2, which addresses body image from a positive perspective; and finally the EAT-26, due to its more sensitive nature. The total estimated completion time will be 25–35 minutes.

For both domains—exercise and eating—the Relative Autonomy Index (RAI) will be calculated. The RAI is a composite score reflecting the degree of self-determination in motivation according to Self-Determination Theory. It is derived by weighting the mean scores of different behavioral regulation types, assigning negative values to less autonomous forms of motivation (amotivation, external and introjected regulation) and positive values to more self-determined forms (identified, integrated, and intrinsic regulation). Exercise-related RAI scores will be calculated from the BREQ-3, and eating-related RAI scores from the REBS. Higher RAI values indicate more autonomous motivation, whereas lower values reflect controlled or amotivated regulation.

3.5. STATISTICAL ANALYSIS AND STUDY VARIABLES

The primary variables will be motivation toward exercise and motivation toward eating, assessed via the BREQ-3 and REBS, respectively, with the Relative Autonomy Index (RAI) calculated as a continuous indicator of self-determination.

Mediating variables will include basic psychological needs (BPNSFS). Secondary outcomes will be physical activity level (IPAQ-short), eating disorder risk (EAT-26), and positive body appreciation (BAS-2).

Statistical analyses will be conducted using RStudio (Version 2025.09.1+401, Posit Software). Descriptive analyses will be performed using measures of central tendency and dispersion, depending on whether variables meet parametric assumptions. To examine intervention and follow-up effects, linear mixed-effects models will be applied using the *lme4* package, including group, time, and their interaction as fixed effects, with random intercepts for participants.

To explore mediation effects of basic psychological needs, the *mediation* package will be used by fitting models for both mediators and outcomes, and estimating indirect effects via bootstrapping. Secondary variables will be analyzed as additional outcomes within mixed-effects models. For non-parametric variables, robust mixed models and bootstrap-based mediation analyses will be applied. Statistical significance will be set at p < .05.

**4. ETHICAL AND CONFIDENTIALITY CONSIDERATIONS**

The study will adhere to the principles of the Declaration of Helsinki (2013) and comply with the General Data Protection Regulation (EU 2016/679) and Organic Law 3/2018 on Personal Data Protection and Guarantee of Digital Rights (LOPDGDD). Participants will sign an informed consent form and receive an information sheet about the project. Participation is voluntary and involves no direct financial incentives. No significant physical or psychological risks are anticipated.

Data will be coded to ensure confidentiality and enable individualized feedback to participants. Given the pre-post evaluation design of the intervention, data coding is necessary to link repeated measures. The research team has accredited experience in nutrition, physical exercise, and eating disorders, and has previously published two studies involving this population.

**5. SUMMARY TIMELINE (JANUARY–NOVEMBER 2026)**

| **Phase** | **Activity** | **Period** |
| --- | --- | --- |
| 1 | Preparation of materials and training of the research team | January–February 2026 |
| 2 | Recruitment and baseline assessment (T0) | 10–28 February 2026 |
| 3 | Intervention (6 workshops, 60 min each) | 3 March–21 April 2026 (Easter break: 28 March–6 April) |
| 4 | Post-intervention assessment (T1) | 22–30 April 2026 |
| 5 | Final follow-up (T2) | Late October 2026 |
| 6 | Data analysis and final reporting | November–December 2026 |

**6. REFERENCIAS**

Cardella, G. M., Hernández-Sánchez, B. R., & Sánchez-García, J. C. (2020). Basic psychological needs as a motivational competence: Examining validity and measurement invariance of Spanish BPNSF scale. Sustainability, 12(13), 5422. <https://doi.org/10.3390/su12135422>

Deci, E. L., & Ryan, R. M. (2000). The “what” and “why” of goal pursuits: Human needs and the self-determination of behavior. Psychological Inquiry, 11(4), 227–268. <https://doi.org/10.1207/S15327965PLI1104_01>

Deforche, B., Van Dyck, D., Deliens, T., & De Bourdeaudhuij, I. (2015). Changes in weight, physical activity, sedentary behaviour and dietary intake during the transition to higher education: A prospective study. International Journal of Behavioral Nutrition and Physical Activity, 12, 16. <https://doi.org/10.1186/s12966-015-0173-9>

Diehl, K., Fuchs, A. K., Rathmann, K., & Hilger-Kolb, J. (2018). Students' Motivation for Sport Activity and Participation in University Sports: A Mixed-Methods Study. BioMed research international, 2018, 9524861. <https://doi.org/10.1155/2018/9524861>

Finlayson, G., Cecil, J., Higgs, S., Hill, A., & Hetherington, M. (2012). Susceptibility to weight gain: Eating behaviour traits and physical activity as predictors of weight gain during the first year of university. Appetite, 58(3), 1091–1098. <https://doi.org/10.1016/j.appet.2012.03.003>

González-Cutre, D., Sicilia, Á., & Fernández, A. (2010). Hacia una mayor comprensión de la motivación en el ejercicio físico: Medición de la regulación integrada en el contexto español. Psicothema, 22(4), 841–847.

Jáuregui Lobera, I., & Bolaños Ríos, P. (2011). Spanish version of the Body Appreciation Scale (BAS) for adolescents. *The Spanish journal of psychology*, *14*(1), 411–420. <https://doi.org/10.5209/rev_sjop.2011.v14.n1.37>

Pelletier, L. G., Dion, S. C., Slovinec-D'Angelo, M., & Reid, R. (2004). Why do you regulate what you eat? Relationships between forms of regulation, eating behaviors, sustained dietary behavior change, and psychological adjustment. *Motivation and Emotion, 28*(3), 245–277. [https://doi.org/10.1023/B:MOEM.0000040154.40922.14](https://psycnet.apa.org/doi/10.1023/B:MOEM.0000040154.40922.14)

Rivas, T., Bersabé, R., Jiménez, M., & Berrocal, C. (2010). The Eating Attitudes Test (EAT-26): reliability and validity in Spanish female samples. *The Spanish journal of psychology*, *13*(2), 1044–1056. <https://doi.org/10.1017/s1138741600002687>

Rodríguez-Muñoz, S., Corella, C., Abarca-Sos, A., & Zaragoza, J. (2017). Validation of three short physical activity questionnaires with accelerometers among university students in Spain. The Journal of sports medicine and physical fitness, 57(12), 1660–1668. <https://doi.org/10.23736/S0022-4707.17.06665-8>

Teixeira, P. J., Marques, M. M., Silva, M. N., Brunet, J., Duda, J. L., Haerens, L., La Guardia, J., Lindwall, M., Lonsdale, C., Markland, D., Michie, S., Moller, A. C., Ntoumanis, N., Patrick, H., Reeve, J., Ryan, R. M., Sebire, S. J., Standage, M., Vansteenkiste, M., . . . Hagger, M. S. (2020). A classification of motivation and behavior change techniques used in self-determination theory-based interventions in health contexts. *Motivation Science, 6*(4), 438–455. [https://doi.org/10.1037/mot0000172](https://psycnet.apa.org/doi/10.1037/mot0000172)

Weman-Josefsson, K., Lindwall, M., & Ivarsson, A. (2015). Need satisfaction, motivational regulations and exercise: Moderation and mediation effects. International Journal of Behavioral Nutrition and Physical Activity, 12, 67. <https://doi.org/10.1186/s12966-015-0226-0>

**PARTICIPANT INFORMATION SHEET FOR RESEARCH STUDY**

**Invitation to participate**

This document aims to provide you with information about the research project entitled “Effect of a Psychoeducational Intervention Based on Self-Determination Theory on Motivation for Physical Exercise and Healthy Eating in Female Health Sciences University Students”, in which you are invited to participate. This study is conducted in accordance with the principles of the Declaration of Helsinki and with standards of good research practice. It has also been approved by the Ethics Committee of the Francisco de Vitoria University.

If you decide to authorize your participation, you should receive personalized information from the researcher, read this document carefully, and ask any questions you may need in order to fully understand the study. You may discuss this document with others and take as much time as you need to decide whether or not to participate.

Participation in this study is entirely voluntary. You may choose not to participate or, if you agree to do so, you may withdraw your consent at any time without providing justification.

**Study objectives**

The aim of this study is to evaluate the effect of a group-based psychoeducational intervention consisting of six workshops on motivation toward physical exercise and healthy eating in female Health Sciences university students. The intervention is grounded in Self-Determination Theory and seeks to foster knowledge, reflection, and the development of personal competencies that promote autonomous motivation—motivation that arises from personal interest and commitment. Through participatory activities and tailored content, participants are encouraged to explore their own motivations and acquire tools to adopt healthier lifestyles in a sustainable way.

Why have I been invited to participate? What will I be asked to do?

This study targets female students from the Faculty of Health Sciences at the Francisco de Vitoria University. This population has been selected because it represents a particularly relevant group for the promotion of healthy habits. University students are in a transitional stage toward adulthood during which behavioral patterns are consolidated and may persist over time. Furthermore, as future health professionals, their commitment to healthy lifestyles may have a positive impact both personally and professionally.

If you agree to participate, you will be invited to take part in a group psychoeducational intervention consisting of six workshops. These workshops aim to promote knowledge, reflection, and the development of personal skills that foster autonomous motivation toward physical exercise and healthy eating. You will also be asked to complete a set of questionnaires before and after the intervention in order to assess your motivation and other aspects related to your lifestyle habits.

**Risks and benefits**

Participation in this study does not entail health risks. The intervention consists of psychoeducational workshops focused on reflection, knowledge, and personal development related to physical activity and healthy eating. No demanding physical activities or direct dietary changes will be required, and no adverse effects are expected.

Regarding benefits, the workshops are designed to foster more autonomous motivation for physical exercise and healthy eating, which may facilitate the adoption of sustainable and positive habits in the long term. Participants will receive up-to-date, evidence-based health information, which may help them make more informed decisions. Additionally, skills such as self-regulation, self-awareness, and self-esteem—fundamental to psychological well-being—will be addressed. The intervention also aims to increase awareness of the importance of adopting healthy lifestyles from an educational and preventive perspective.

Through the study questionnaires, aspects such as exercise motivation, regulation of eating behavior, satisfaction of basic psychological needs, physical activity level, body appreciation, and potential risky eating attitudes will be explored. This may allow participants to gain greater self-knowledge and reflect on their own habits.

**Voluntary participation**

Participation in this study is entirely voluntary. You may choose not to participate or withdraw at any time without any consequences or negative impact on your relationship with the research team. You will be informed of any relevant new information that may influence your decision to continue participating. Participation in this study does not provide academic benefits or financial compensation.

**Confidentiality of information**

This study is subject to Law 14/2007 on Biomedical Research.

The processing, communication, and transfer of your data will be carried out in accordance with Regulation (EU) 2016/679 of the European Parliament and of the Council of 27 April 2016 on the protection of natural persons with regard to the processing of personal data and on the free movement of such data (GDPR), as well as with Organic Law 3/2018 of 5 December on the Protection of Personal Data and the Guarantee of Digital Rights (LOPDGDD). At any time, you may access your data, object to their processing, rectify or delete them by contacting the Data Protection Officer (dpd@ufv.es). Only the researchers involved in the study will have access to such data, and it is guaranteed that, in the event that the results of the study are published, the identity of the participant will remain confidential. All research data will be coded at all times, and the research team will ensure that personal data and study data are kept separately. The rights of access, rectification, deletion, and objection regarding the study data are guaranteed via email to the principal investigator (sonia.garciamerino@ufv.es). In addition, any possible processing of your health data must be expressly authorized through informed consent.

Contact with the researchers

The researchers responsible for the study are Sonia García Merino, Germán Díaz Ureña, Patricia Ruiz Bravo, Bárbara Rodríguez Rodríguez, Cristina Blanco Llamero, and Noelia Belando. To request any additional information or to resolve questions or doubts related to this study, you may contact the researchers via email at sonia.garciamerino@ufv.es; german.diaz@ufv.es; p.ruiz.prof@ufv.es; br.rodriguez.prof@ufv.es; cristina.blanco@ufv.es; belandonoelia@uniovi.es, or in person.

In any case, should you have doubts about granting consent to participate in the study, the participant may consult any individuals they consider appropriate (family members, friends, family physician, lecturers, etc.).

Date: Date:
Participant’s signature	Principal Investigator’s signature

**INFORMED CONSENT TO PARTICIPATE IN THE STUDY**

**“Efecto de una intervención psicoeducativa basada en la Teoría de la Autodeterminación sobre la motivación hacia el ejercicio físico y la alimentación saludable en mujeres universitarias de Ciencias de la Salud”**

**“Effect of a Psychoeducational Intervention Based on Self-Determination Theory on Motivation for Physical Exercise and Healthy Eating in Female Health Sciences University Students”**

I, Mr./Ms. ____________________________________________________________________, have read the information sheet, have understood the explanations provided to me, as well as the possible complications and risks. Therefore, I declare that I have been adequately informed in accordance with Article 4 of Law 41/2002 of November 14, and I give my consent to participate in the research study and for my data to be used for the publications explained to me in the information sheet.

In..........................................................., on the ………. of .................................................. of 20…..

Signed:
ID Number:

**Basic information regarding the protection of your personal data:**

The data controller responsible for processing your data is the Francisco de Vitoria University (UFV).
The purpose of the data processing is to manage your participation in the project *“Effect of a Psychoeducational Intervention Based on Self-Determination Theory on Motivation for Physical Exercise and Healthy Eating in Female Health Sciences University Students”*, as described in this document.

The legal basis for data processing is the consent of the data subject, provided by ticking the relevant boxes. Your personal data will not be disclosed to third parties. The data will be retained for as long as necessary for the scientific research purposes pursued by the aforementioned research project, and once the research project has ended, the data may be anonymized due to the scientific interest of the study. Participation in this study is completely voluntary. The participant may withdraw at any time without any consequences, provided that this is previously communicated to the principal investigator.

This study complies with Law 14/2007 on Biomedical Research, which guarantees the protection of participants’ rights. The processing of personal data will be carried out in compliance with Regulation (EU) 2016/679 (GDPR) and Organic Law 3/2018 on the Protection of Personal Data and the Guarantee of Digital Rights (LOPDGDD), ensuring confidentiality, security, and responsible use. You may exercise your rights of access, rectification, erasure, objection, restriction of processing, and data portability by submitting a written request to the General Secretariat of the Francisco de Vitoria University, Ctra. M-515 Pozuelo–Majadahonda Km. 1.800; 28223, Pozuelo de Alarcón (Madrid), or by email to dpd@ufv.es.

If at any time you provide us with data relating to third parties, you are hereby informed that you are obliged to inform those individuals of the content of this clause. The participant is informed that they may not provide information that identifies third parties without their prior written consent.

You may consult extended information at the following website: https://www.ufv.es/politica-de-privacidad

☐ Yes   ☐ No
I accept and authorize the Francisco de Vitoria University to process my data for the purpose of participating in the research project and to send me, by any means including electronic means (by way of example, but not limited to, email), communications related to my participation in the aforementioned research project.

**Special consent regarding the processing of health data:**
In the referenced research project, data relating to your health may be processed. In order for such data to be used for scientific research purposes, you must authorize the Francisco de Vitoria University to carry out such processing by providing your explicit consent by ticking the following box.

☐ Yes   ☐ No
I accept and authorize the Francisco de Vitoria University to process my health data obtained through voluntary participation in this research project.

Date: Date:

Participant’s signature	Principal Investigator’s signature
