# Supplementary material for: Study protocol for a randomized controlled trial of a self-determination theory–based psychoeducational intervention to enhance autonomous motivation for exercise in female university students
Source: PLoS One. 2026 Aug 7;21(8):e0355374. doi: 10.1371/journal.pone.0355374 (PMC13450776; doi:10.1371/journal.pone.0355374)
Supplement: Supplementary file 6 [file pone.0355374.s005.docx]

**MEMORIA DEL PROYECTO PARA COMITÉ ÉTICO**

Título del estudio: Efecto de una intervención psicoeducativa basada en la *Teoría de la Autodeterminación* sobre la motivación hacia el ejercicio físico y la alimentación saludable en mujeres universitarias de Ciencias de la Salud.

Investigador/a principal: Dra. Sonia García Merino/Co-director: Germán Díaz Ureña

Centro / Facultad: Facultad de Ciencias de la Salud

Correo electrónico: sonia.garciamerino@ufv.es

Duración prevista: Enero 2026 – Diciembre 2026

**1. INTRODUCCIÓN: JUSTIFICACIÓN Y CONTEXTO DEL ESTUDIO**

Los primeros años de la universidad constituyen una etapa de transición que puede alterar los hábitos de alimentación y actividad física de los jóvenes. Diversos estudios longitudinales han mostrado que este periodo se asocia con un descenso de la actividad física y una mayor irregularidad en la dieta (Deforche et al., 2015; Finlayson et al., 2012). En este contexto, las motivaciones que impulsan las conductas saludables adquieren especial relevancia entre la población universitaria, es frecuente que la práctica de ejercicio y el control alimentario respondan a fines estéticos o sociales más que a razones de bienestar o autocuidado (Diehl et al., 2018). Comprender qué tipo de motivaciones sustentan estas conductas resulta fundamental, ya que el modo en que las personas se relacionan con el ejercicio y la alimentación influye directamente en la calidad de sus hábitos y en su bienestar psicológico.

Desde esta perspectiva, la Teoría de la Autodeterminación (SDT; Deci & Ryan, 2000) ofrece un marco explicativo sólido para analizar los diferentes tipos de motivación hacia las conductas saludables. Según esta teoría, la calidad de la motivación autónoma frente a controlada determina la adherencia y el impacto psicológico de dichas conductas. Cuando las personas actúan movidas por interés y elección personal (motivación autónoma), tienden a mantener los hábitos de forma más sostenible y con mayor bienestar emocional, mientras que las motivaciones controladas (culpa, presión o deseo de aprobación) se asocian con menor satisfacción y con el riesgo de conductas desadaptativas. Intervenciones breves basadas en la SDT han demostrado mejorar la motivación autónoma y los hábitos saludables en universitarios (Teixeira et al., 2020; Weman-Josefsson et al., 2015).

En este marco teórico se sitúa la línea de investigación desarrollada por nuestro grupo, cuyos resultados previos han permitido avanzar en la comprensión de los factores motivacionales vinculados con la imagen corporal y las conductas de salud en población universitaria. En un primer trabajo, publicado en Frontiers in Psychology (2025), se observaron diferencias por sexo en la percepción corporal y en el riesgo de trastornos de la conducta alimentaria (TCA). Posteriormente, un segundo estudio publicado en Healthcare (2025), centrado en mujeres universitarias, mostró una asociación positiva entre el nivel de actividad física y las puntuaciones del EAT-26, aunque sin alcanzar valores patológicos. Estos hallazgos sugieren que una parte del ejercicio físico podría estar guiado por motivos controlados, como la búsqueda estética o la presión social, en lugar de por motivos autónomos relacionados con la salud y el bienestar. A partir de estos resultados, se propone un tercer estudio orientado a evaluar el efecto de una intervención psicoeducativa basada en la Teoría de la Autodeterminación, cuyo objetivo es promover una motivación más autónoma hacia el ejercicio y la alimentación, favoreciendo hábitos saludables y previniendo comportamientos de riesgo relacionados con la imagen corporal.

**2. OBJETIVOS**

Objetivo general:

Evaluar el efecto de una intervención psicoeducativa grupal basada en la Teoría de la Autodeterminación sobre la motivación hacia el ejercicio físico y la alimentación saludable en mujeres universitarias de Ciencias de la Salud.

Objetivos específicos:

Analizar los cambios en la motivación hacia el ejercicio (BREQ-3; RAI) y hacia la alimentación (REBS; RAI) tras la intervención.

Examinar el efecto sobre la satisfacción y frustración de las necesidades psicológicas básicas (BPNSFS).

Evaluar el impacto en el nivel de actividad física (IPAQ-short), el riesgo de TCA (EAT-26) y la insatisfacción corporal (BAS-2;Body Appreciation Scale-2).

Determinar si los cambios motivacionales median la relación entre la intervención y las mejoras en los hábitos de ejercicio y alimentación.

**3. METODOLOGÍA**

3.1. DISEÑO

Se realizará un ensayo controlado aleatorizado con tres mediciones: preintervención (T0), postintervención (T1) y seguimiento a los seis meses (T2). Tras la evaluación basal, se llevará a cabo la aleatorización de la muestra mediante muestreo aleatorio simple, utilizando un generador de números aleatorios en RStudio (Versión 2025.09.1+401, Posit Software). Las participantes serán asignadas 1:1 a un grupo intervención o a un grupo control en lista de espera.

La intervención constará de seis talleres grupales presenciales de 60 minutos, realizados entre marzo y abril de 2026, centrados en ejercicio saludable, alimentación saludable y motivación autónoma. El grupo control no recibirá intervención durante el estudio, pero se le ofrecerá el programa completo al finalizar el seguimiento (T2).

3.2. PARTICIPANTES Y CRITERIOS DE SELECCIÓN

La población diana estará compuesta por estudiantes de los grados en Ciencias de la Actividad Física y del Deporte, Fisioterapia, Nutrición Humana y Dietética, y Enfermería de la Universidad Francisco de Vitoria.

La muestra incluirá mujeres de entre 18 y 25 años, matriculadas en alguno de los grados mencionados, que acepten participar de forma voluntaria mediante la firma del consentimiento informado.

Se excluirán aquellas participantes que no completen los cuestionarios en alguno de los momentos de medición (T0 o T1), las que no asistan al menos al 75 % de las sesiones programadas en el grupo intervención y las que presenten diagnóstico actual o previo de TCA o TCAN.

3.3. TAMAÑO MUESTRAL

El tamaño muestral se calculó a priori mediante la herramienta G*Power (versión 2.9, Universidad de Düsseldorf), utilizando un diseño de F tests – ANOVA: Repeated measures, within-between interaction.

Los parámetros considerados fueron: tamaño del efecto esperado pequeño (η²p = 0.01), nivel de significación (α = 0.05), potencia (1–β = 0.8), dos grupos de comparación, tres medidas repetidas por sujeto, correlación entre medidas (r = 0.5) y corrección de no esfericidad de 1.

Según estos parámetros, el tamaño muestral requerido fue de 162 participantes (81 por grupo). Para prever una pérdida experimental del 20 %, el tamaño de muestra objetivo se fijó en 194 participantes.

3.4. PROCEDIMIENTOS DE APLICACIÓN

Las herramientas empleadas incluyen los siguientes cuestionarios y escalas:

- BREQ-3 (González-Cutre et al., 2010)
- REBS (Pelletier et al., 2004)
- BPNSFS (Cardella et al., 2020)
- IPAQ-short (Rodríguez-Muñoz et al., 2017)
- EAT-26 (Rivas et al., 2010)
- BAS-2 Body Appreciation Scale-2 (Jauregui Lobera et al., 2011)

Los cuestionarios se administrarán en un orden diseñado para minimizar sesgos y preservar el bienestar de las participantes: primero el BPNSFS, de contenido general y emocionalmente neutro; seguido del BREQ-3 y el REBS, que evalúan la motivación hacia el ejercicio y la alimentación; a continuación, el IPAQ-short, como medida conductual intermedia; luego el BAS-2, que aborda la imagen corporal desde una perspectiva positiva; y finalmente el EAT-26, debido a su carácter más sensible. La duración total estimada será de 25 a 35 minutos.

Para ambos dominios, ejercicio y alimentación, se calculará el Índice de Autonomía Relativa (RAI), una medida compuesta que refleja el grado de autodeterminación en la motivación según la Teoría de la Autodeterminación. El RAI se obtiene ponderando las puntuaciones medias de las distintas formas de regulación del comportamiento, asignando valores negativos a las motivaciones menos autónomas (amotivación, regulación externa e introyectada) y positivos a las más autodeterminadas (identificada, integrada e intrínseca). En el caso del ejercicio, las puntuaciones se derivarán del cuestionario BREQ-3, y para la alimentación de la escala REBS. Valores más altos del RAI indican una motivación más autónoma, mientras que valores bajos reflejan una motivación controlada o amotivada.

3.5. ANÁLISIS ESTADÍSTICO Y VARIABLES DE ESTUDIO

Las variables principales serán la motivación hacia el ejercicio y la motivación hacia la alimentación, evaluadas mediante el BREQ-3 y el REBS, respectivamente. En ambos casos se calculará el Índice de Autonomía Relativa (RAI) como indicador continuo del grado de autodeterminación.
Las variables mediadoras incluirán las necesidades psicológicas básicas (BPNSFS) y las variables secundarias serán la actividad física (IPAQ-short), el riesgo de TCA (EAT-26) y la apreciación corporal positiva (BAS-2).

El análisis estadístico se realizará en RStudio (Versión 2025.09.1+401, Posit Software). Se efectuará un análisis descriptivo de los valores de tendencia central y dispersión, en función de si los valores son paramétricos o no paramétricos. Para evaluar los efectos de la intervención y el seguimiento se emplearán modelos lineales mixtos mediante el paquete lme4, considerando el factor grupo, el factor tiempo y su interacción, con intercepto aleatorio por sujeto.

Para explorar la mediación de las necesidades psicológicas básicas, se utilizará el paquete mediation, ajustando modelos para el mediador y el resultado (outcome), y estimando los efectos indirectos mediante bootstrapping. Las variables secundarias se analizarán como outcomes adicionales en modelos mixtos. En caso de variables no paramétricas, se aplicarán modelos mixtos robustos y mediación basada en bootstrap. Se establecerá un nivel de significación de p < .05.

**4. ASPECTOS ÉTICOS Y DE CONFIDENCIALIDAD**

El estudio seguirá los principios de la Declaración de Helsinki (2013) y el Reglamento General de Protección de Datos (UE 2016/679) y Ley Orgánica 3/2018 de protección de datos personales y garantía de los derechos digitales (LOPDGDD). Las participantes firmarán consentimiento informado y se les proporcionará una hoja de información sobre el proyecto. La participación es voluntaria, sin incentivos económicos directos. No se prevén riesgos físicos ni psicológicos significativos. Los datos serán codificados para la protección de datos y para poder dar, de esa manera, feedback individualizado a las participantes, además se trata de una intervención con evaluación prepos por lo que resulta necesario para poder asociar los datos El equipo investigador cuenta con experiencia acreditada en nutrición, ejercicio físico y TCA, y ha publicado dos estudios previos sobre esta población.

**5. CRONOGRAMA RESUMIDO (ENERO–NOVIEMBRE 2026)**

| Fase | Actividad | Periodo |
| --- | --- | --- |
| 1 | Preparación de materiales y formación del equipo de trabajo. | Enero–Febrero 2026 |
| 2 | Reclutamiento y evaluación basal (T0) | 10–28 Febrero 2026 |
| 3 | Intervención (6 talleres de 60 min) | 3 Marzo–21 Abril 2026 (pausa Semana Santa 28 Mar.–6 Abr.) |
| 4 | Evaluación post-intervención (T1) | 22–30 Abril 2026 |
| 5 | Seguimiento final (T2) | Final de Octubre 2026 |
| 6 | Análisis y redacción final | Noviembre-Diciembre 2026 |

**6. REFERENCIAS**

Cardella, G. M., Hernández-Sánchez, B. R., & Sánchez-García, J. C. (2020). Basic psychological needs as a motivational competence: Examining validity and measurement invariance of Spanish BPNSF scale. Sustainability, 12(13), 5422. <https://doi.org/10.3390/su12135422>

Deci, E. L., & Ryan, R. M. (2000). The “what” and “why” of goal pursuits: Human needs and the self-determination of behavior. Psychological Inquiry, 11(4), 227–268. <https://doi.org/10.1207/S15327965PLI1104_01>

Deforche, B., Van Dyck, D., Deliens, T., & De Bourdeaudhuij, I. (2015). Changes in weight, physical activity, sedentary behaviour and dietary intake during the transition to higher education: A prospective study. International Journal of Behavioral Nutrition and Physical Activity, 12, 16. <https://doi.org/10.1186/s12966-015-0173-9>

Diehl, K., Fuchs, A. K., Rathmann, K., & Hilger-Kolb, J. (2018). Students' Motivation for Sport Activity and Participation in University Sports: A Mixed-Methods Study. BioMed research international, 2018, 9524861. <https://doi.org/10.1155/2018/9524861>

Finlayson, G., Cecil, J., Higgs, S., Hill, A., & Hetherington, M. (2012). Susceptibility to weight gain: Eating behaviour traits and physical activity as predictors of weight gain during the first year of university. Appetite, 58(3), 1091–1098. <https://doi.org/10.1016/j.appet.2012.03.003>

González-Cutre, D., Sicilia, Á., & Fernández, A. (2010). Hacia una mayor comprensión de la motivación en el ejercicio físico: Medición de la regulación integrada en el contexto español. Psicothema, 22(4), 841–847.

Jáuregui Lobera, I., & Bolaños Ríos, P. (2011). Spanish version of the Body Appreciation Scale (BAS) for adolescents. *The Spanish journal of psychology*, *14*(1), 411–420. <https://doi.org/10.5209/rev_sjop.2011.v14.n1.37>

Pelletier, L. G., Dion, S. C., Slovinec-D'Angelo, M., & Reid, R. (2004). Why do you regulate what you eat? Relationships between forms of regulation, eating behaviors, sustained dietary behavior change, and psychological adjustment. *Motivation and Emotion, 28*(3), 245–277. [https://doi.org/10.1023/B:MOEM.0000040154.40922.14](https://psycnet.apa.org/doi/10.1023/B:MOEM.0000040154.40922.14)

Rivas, T., Bersabé, R., Jiménez, M., & Berrocal, C. (2010). The Eating Attitudes Test (EAT-26): reliability and validity in Spanish female samples. *The Spanish journal of psychology*, *13*(2), 1044–1056. <https://doi.org/10.1017/s1138741600002687>

Rodríguez-Muñoz, S., Corella, C., Abarca-Sos, A., & Zaragoza, J. (2017). Validation of three short physical activity questionnaires with accelerometers among university students in Spain. The Journal of sports medicine and physical fitness, 57(12), 1660–1668. <https://doi.org/10.23736/S0022-4707.17.06665-8>

Teixeira, P. J., Marques, M. M., Silva, M. N., Brunet, J., Duda, J. L., Haerens, L., La Guardia, J., Lindwall, M., Lonsdale, C., Markland, D., Michie, S., Moller, A. C., Ntoumanis, N., Patrick, H., Reeve, J., Ryan, R. M., Sebire, S. J., Standage, M., Vansteenkiste, M., . . . Hagger, M. S. (2020). A classification of motivation and behavior change techniques used in self-determination theory-based interventions in health contexts. *Motivation Science, 6*(4), 438–455. [https://doi.org/10.1037/mot0000172](https://psycnet.apa.org/doi/10.1037/mot0000172)

Weman-Josefsson, K., Lindwall, M., & Ivarsson, A. (2015). Need satisfaction, motivational regulations and exercise: Moderation and mediation effects. International Journal of Behavioral Nutrition and Physical Activity, 12, 67. <https://doi.org/10.1186/s12966-015-0226-0>

**HOJA DE INFORMACIÓN AL PARTICIPANTE PARA ESTUDIO DE INVESTIGACIÓN.**

**Invitación a participar**

Este documento tiene por objeto ofrecerle información sobre el proyecto: **“Efecto de una intervención psicoeducativa basada en la Teoría de la Autodeterminación sobre la motivación hacia el ejercicio físico y la alimentación saludable en mujeres universitarias de Ciencias de la Salud”**, en el que se le invita a participar. Este estudio se lleva a cabo con respeto a los principios enunciados en la declaración del Helsinki y a las normas de buena práctica en investigación. Además, ha sido aprobado por el Comité ético de la Universidad Francisco de Vitoria.

Si decide autorizar su participación en el mismo, debe recibir información personalizada del investigador, leer antes este documento y hacer todas las preguntas que precise para comprender los detalles sobre este. Este documento puede consultarlo con otras personas y tomarse el tiempo que necesite para decidir si autoriza su participación o no.

La participación en este estudio es completamente voluntaria. Puede decidir no participar o, si acepta hacerlo, cambiar de parecer retirando el consentimiento en cualquier momento sin dar explicaciones.

**Objetivos del estudio**

Este estudio tiene como objetivo evaluar el efecto de una intervención psicoeducativa grupal, compuesta por seis talleres, sobre la motivación hacia el ejercicio físico y la alimentación saludable en mujeres universitarias de Ciencias de la Salud. La intervención está basada en la Teoría de la Autodeterminación y busca fomentar el conocimiento, la reflexión y el desarrollo de competencias personales que promuevan una motivación autónoma, es decir, aquella que nace del interés y el compromiso personal. A través de dinámicas participativas y contenidos adaptados, se pretende que las participantes exploren sus propias motivaciones y adquieran herramientas para adoptar hábitos de vida más saludables de forma sostenible.

**¿Por qué me ofrecen participar a mí? ¿Qué tendré que hacer?**

Este estudio está dirigido a mujeres estudiantes de la Facultad de Ciencias de la Salud de la Universidad Francisco de Vitoria. Se ha elegido esta población porque representa un grupo especialmente relevante para la promoción de hábitos saludables. Las estudiantes universitarias se encuentran en una etapa de transición hacia la vida adulta en la que se consolidan patrones de comportamiento que pueden mantenerse a lo largo del tiempo. Además, al tratarse de futuras profesionales del ámbito de la salud, su implicación en el desarrollo de estilos de vida saludables puede tener un impacto positivo tanto a nivel personal como profesional.

Si acepta participar, se le invitará a formar parte de una intervención psicoeducativa grupal compuesta por seis talleres. Estos talleres están diseñados para fomentar el conocimiento, la reflexión y el desarrollo de competencias personales que promuevan una motivación autónoma hacia el ejercicio físico y la alimentación saludable. Además, se le pedirá que complete una serie de cuestionarios antes y después de la intervención, con el fin de evaluar su motivación y otros aspectos relacionados con sus hábitos de vida.

**Riesgos y beneficios**

La participación en este estudio no conlleva riesgos para la salud. La intervención consiste en una serie de talleres psicoeducativos centrados en la reflexión, el conocimiento y el desarrollo personal en relación con la actividad física y la alimentación saludable. No se realizarán actividades físicas exigentes ni se propondrán cambios dietéticos directos, por lo que no se espera que la participación tenga efectos adversos.

En cuanto a los beneficios, los talleres están diseñados para fomentar una motivación más autónoma hacia el ejercicio físico y la alimentación saludable, lo que puede favorecer la adopción de hábitos sostenibles y positivos a largo plazo. Las participantes recibirán información actualizada y útil sobre salud, basada en recomendaciones científicas, lo que puede mejorar su capacidad para tomar decisiones informadas. Además, se trabajarán habilidades como la autorregulación, el autoconocimiento y la autoestima, fundamentales para el bienestar psicológico. También se promoverá una mayor conciencia sobre la importancia de adoptar estilos de vida saludables desde una perspectiva educativa y preventiva.

A través de los cuestionarios incluidos en el estudio, se explorarán aspectos como la motivación hacia el ejercicio, la regulación de la conducta alimentaria, la satisfacción de necesidades psicológicas básicas, el nivel de actividad física, la apreciación corporal y posibles actitudes alimentarias de riesgo. Esta información permitirá a las participantes conocerse mejor y reflexionar sobre sus propios hábitos.

**Voluntariedad de la participación**

La participación en este estudio es completamente voluntaria. Usted puede decidir no participar o retirarse en cualquier momento sin que ello implique ningún tipo de perjuicio ni afecte su relación con el equipo investigador.

Durante el desarrollo del estudio, se le informará de cualquier novedad relevante que pueda influir en su decisión de continuar participando. Es importante destacar que la participación en este estudio no conlleva ningún tipo de beneficio académico ni compensación económica.

**Confidencialidad de la información**

El presente estudio está sometido a la Ley 14/2007 de investigación biomédica.

El tratamiento, comunicación y cesión de sus datos se hará conforme a lo dispuesto por el Reglamento (UE) 2016/679 del Parlamento Europeo y del Consejo de 27 de abril de 2016 relativo a la protección de las personas físicas en lo que respecta al tratamiento de datos personales y a la libre circulación de estos datos (RGPD) y en la Ley Orgánica 3/2018, de 5 de diciembre, de Protección de Datos de Carácter Personal y garantía de los derechos digitales (LOPDGDD). En todo momento, podrá acceder a sus datos, oponerse, corregirlos o cancelarlos, se debe poner en contacto con la delegada de Protección de Datos ([dpd@ufv.es](mailto:dpd@ufv.es)). Únicamente los investigadores del estudio podrán tener acceso a dichos datos, y se garantiza que, en caso de publicación de los resultados del estudio, la identidad del sujeto será confidencial. Todos los datos de la investigación estarán codificados en todo momento y el equipo investigador garantizará que los datos personales y del estudio se mantienen separados. Se garantiza el derecho de acceso, rectificación, cancelación y oposición a los datos del estudio mediante un correo electrónico al investigador principal ([sonia.garciamerino@ufv.es](mailto:sonia.garciamerino@ufv.es)). Además, el posible tratamiento de sus datos de salud deberá ser expresamente autorizado a través del consentimiento informado.

**Contacto con investigadores**

Los investigadores responsables del estudio son Sonia García Merino, Germán Díaz Ureña, Patricia Ruiz Bravo, Bárbara Rodríguez Rodríguez, Cristina Blanco Llamero y Noelia Belando. Para solicitar cualquier información adicional o resolver dudas y preguntas relativas a este estudio puede contactar con los investigadores, a través del correo electrónico [sonia.garciamerino@ufv.es](mailto:sonia.garciamerino@ufv.es); [german.diaz@ufv.es](mailto:german.diaz@ufv.es); [p.ruiz.prof@ufv.es](mailto:p.ruiz.prof@ufv.es); [br.rodriguez.prof@ufv.es](mailto:br.rodriguez.prof@ufv.es); [cristina.blanco@ufv.es](mailto:cristina.blanco@ufv.es); [belandonoelia@uniovi.es](mailto:belandonoelia@uniovi.es) o personalmente.

De cualquier modo, en caso de tener dudas para otorgar el consentimiento de participación en el estudio, el participante podrá consultar a las personas que considere oportuno (familiares, amigos, médico de familia, profesores, …).

Fecha: Fecha:

Firma del participante	Firma del investigador principal

**CONSENTIMIENTO INFORMADO DE PARTICIPACIÓN EN EL ESTUDIO “Efecto de una intervención psicoeducativa basada en la Teoría de la Autodeterminación sobre la motivación hacia el ejercicio físico y la alimentación saludable en mujeres universitarias de Ciencias de la Salud”**

Yo, D/_____________________________________________________________________________________, he leído la hoja de información, he comprendido las explicaciones que se me han facilitado, así como las posibles complicaciones y riesgos. Por tanto, declaro estar correctamente informado/a según dispone el artículo 4 de la Ley 41/2002, de 14 de noviembre, y doy mi consentimiento para participar en el estudio de investigación y que mis datos sean utilizados para las publicaciones que se me han explicado en la hoja de información.

En ................................................................................., a………. de........................................................ de 20..…..

Fdo.:

D.N.I.:

**Información básica relativa a la protección de sus datos de carácter personal:**

El responsable del tratamiento de sus datos es la Universidad Francisco de Vitoria (UFV).

La finalidad del tratamiento es gestionar su participación en el proyecto “**Efecto de una intervención psicoeducativa basada en la Teoría de la Autodeterminación sobre la motivación hacia el ejercicio físico y la alimentación saludable en mujeres universitarias de Ciencias de la Salud** " tal y como se describe en este documento.

La legitimación del tratamiento es el consentimiento del interesado marcando las casillas destinadas a tal efecto. Sus datos personales no serán comunicados a terceros. Los datos serán conservados mientras sea necesario para los fines de investigación científica que persigue el proyecto de investigación anteriormente señalado y, una vez finalizado el proyecto de investigación, los datos podrán anonimizarse debido al interés científico del estudio. La participación en este estudio es completamente voluntaria. El participante podrá retirarse en cualquier momento sin que ello implique repercusiones de ningún tipo, siempre que lo comunique previamente al investigador principal.

Este estudio cumple con la Ley 14/2007 de Investigación Biomédica, que garantiza la protección de los derechos de los participantes. El tratamiento de los datos personales se realizará conforme al Reglamento General de Protección de Datos (UE) 2016/679 (RGPD) y a la Ley Orgánica 3/2018, de Protección de Datos Personales y garantía de los derechos digitales (LOPDGDD), asegurando su confidencialidad, seguridad y uso responsable Puede ejercitar sus derechos de acceso, rectificación, supresión, oposición, limitación del tratamiento y portabilidad mediante un escrito dirigido a la Secretaría General de la Universidad Francisco de Vitoria, Ctra. M-515 Pozuelo-Majadahonda Km. 1,800; 28223, Pozuelo de Alarcón (Madrid), o al correo electrónico [dpd@ufv.es](mailto:dpd@ufv.es).

Si en algún momento nos facilita datos de terceras personas, le informamos de que queda obligado a informar al interesado sobre el contenido de esta cláusula. El participante queda informado que no puede facilitar información que identifique a terceros sin obtener su consentimiento previo y por escrito.

Puede consultar la información ampliada en la web <https://www.ufv.es/politica-de-privacidad>

Si	No	Acepto y autorizo a que mis datos sean tratados por la Universidad Francisco de Vitoria, con la finalidad de participar en el proyecto de investigación y para remitirme, por cualquier medio, incluidos los electrónicos (a modo enunciativo, pero no limitativo, correo electrónico), comunicaciones relativas a mi participación en el proyecto de investigación referenciado.

**Consentimiento especial en relación con el tratamiento de datos de salud:** en el proyecto de investigación referenciado se pueden llegar a tratar datos sobre su salud. Para poder utilizarlos con fines de investigación científica, debe autorizar a la Universidad Francisco de Vitoria dicho tratamiento mediante el correspondiente consentimiento explícito marcando la siguiente casilla.

Si  No	 Acepto y autorizo a que mis datos de salud obtenidos a través de la participación voluntaria en este proyecto de investigación sean tratados por la Universidad Francisco de Vitoria.

Fecha: Fecha:

Firma del participante	Firma del investigador principal
