# Supplementary material for: Study protocol for a randomized controlled trial of a self-determination theory–based psychoeducational intervention to enhance autonomous motivation for exercise in female university students
Source: PLoS One. 2026 Aug 7;21(8):e0355374. doi: 10.1371/journal.pone.0355374 (PMC13450776; doi:10.1371/journal.pone.0355374)
Supplement: Supplementary file 7 [file pone.0355374.s008.docx]

**Response to Reviewers**

*Manuscript: A randomized controlled trial of a Self-Determination Theory–based psychoeducational intervention to enhance autonomous motivation for exercise in female university students*

We thank the Editor and both Reviewers for their careful evaluation of the revised manuscript. We are pleased that the reviewers considered the previous comments satisfactorily addressed and that the methodological revisions were considered to strengthen the study protocol. We have carefully considered the remaining comment and revised both the manuscript and the Statistical Analysis Plan (SAP) accordingly.

# Reviewer 1

We thank the reviewer for the positive evaluation of the revised manuscript and for confirming that the previous comments have been satisfactorily addressed.

# Reviewer 2

## Comment 5 — MMRM instead of multiple imputation; GLMM for count/categorical

*“The manuscript and SAP have been revised accordingly. Multiple imputation has*

*been removed as the primary strategy for handling missing data. The primary analysis is now specified as a Mixed Model for Repeated Measures (MMRM) under a Missing At Random (MAR) assumption, incorporating all observed data without imputation. For outcomes that do not satisfy distributional assumptions, appropriate generalized mixed models are specified.*

*I'm happy to see the move to MMRM, However, now that the authors have identified a primary timepoint as T1, MMRM will not work for the primary outcome analysis. If the primary outcome is T1, there will only be two measurements, and MMRM requires a minimum of 3 timepoints to work. MMRM would be optimal if the primary outcome was analyzed at T2. A standard mixed model approach does not account for missingness and so MI is more appropriate if T1 is the primary endpoint.The authors will now need to either change the primary outcome endpoint to T2, or change back to standard mixed model with MI for the primary outcome (SAP and Methods). MMRM/GLMM will still be most appropriate for secondary outcomes at T2.”*

**Response.** We thank the Reviewer for this constructive methodological critique. Following the Reviewer’s recommendations, our team has carefully re-evaluated the core conceptual and investigational goals of this trial. The fundamental purpose of our Self-Determination Theory (SDT)-based psychoeducational program is to foster deep internalization of exercise motives and sustainable behavioral changes. Therefore, from a clinical and behavioral perspective, the long-term maintenance of autonomous motivation at the 6-month follow-up (T2) represents our primary endpoint.

This refinement establishes a highly coherent framework:

- **Methodological Soundness:** By setting T2 as the primary endpoint, the MMRM now evaluates the full evaluation trajectory (T0 as a baseline covariate, T1 as an intermediate secondary milestone, and T2 as the primary endpoint). This satisfies the minimum requirement of 3 timepoints, mathematically validating the MMRM structure and completely preserving its capacity to handle missing data under MAR without requiring prior multiple imputation.
- **Statistical Power Realignment:** We have updated our simulation-based power script in R to align with this change.

We believe this adjustment has significantly strengthened the transparency, reproducibility, and mathematical internal validity of our protocol.

**Changes made:**

Manuscript

-Abstract: page 5, lines 36 and 38-39

-Sample Size: page 8, lines193-194 and 204

-Measures: Primary outcomes: page 15, lines 322

- Statistical Analysis: page 18, lines: 396–398 and 418).

SAP:

3.1, 3.2, 4, 5.2, 5.4, 5.5, 8.2 and 12
