# Supplementary material for: Study protocol for a randomized controlled trial of a self-determination theory–based psychoeducational intervention to enhance autonomous motivation for exercise in female university students
Source: PLoS One. 2026 Aug 7;21(8):e0355374. doi: 10.1371/journal.pone.0355374 (PMC13450776; doi:10.1371/journal.pone.0355374)
Supplement: Peer review history

# Peer review: Decision Letter 0

**DOI:** 10.1371/journal.pone.0355374.r001

**Authors:** Wanli Zang

15 Jun 2026

PONE-D-26-19756

Study protocol for a randomized controlled trial of a self-determination theory–based psychoeducational intervention to enhance autonomous motivation for exercise in female university students

PLOS One

Dear Dr. Ruiz-Bravo,

Thank you for submitting your manuscript to PLOS ONE. After careful consideration, we feel that it has merit but does not fully meet PLOS ONE’s publication criteria as it currently stands. Therefore, we invite you to submit a revised version of the manuscript that addresses the points raised during the review process.

Please submit your revised manuscript by Jul 30 2026 11:59PM. If you will need more time than this to complete your revisions, please reply to this message or contact the journal office at plosone@plos.org. When you're ready to submit your revision, log on to https://www.editorialmanager.com/pone/ and select the 'Submissions Needing Revision' folder to locate your manuscript file.

Please include the following items when submitting your revised manuscript:

- A letter that responds to each point raised by the academic editor and reviewer(s). You should upload this letter as a separate file labeled 'Response to Reviewers'.
- A marked-up copy of your manuscript that highlights changes made to the original version. You should upload this as a separate file labeled 'Revised Manuscript with Track Changes'.
- An unmarked version of your revised paper without tracked changes. You should upload this as a separate file labeled 'Manuscript'.

If you would like to make changes to your financial disclosure, please include your updated statement in your cover letter. Guidelines for resubmitting your figure files are available below the reviewer comments at the end of this letter.

If applicable, we recommend that you deposit your laboratory protocols in protocols.io to enhance the reproducibility of your results. Protocols.io assigns your protocol its own identifier (DOI) so that it can be cited independently in the future. For instructions see: https://journals.plos.org/plosone/s/submission-guidelines#loc-laboratory-protocols. Additionally, PLOS ONE offers an option for publishing peer-reviewed Lab Protocol articles, which describe protocols hosted on protocols.io. Read more information on sharing protocols at https://plos.org/protocols?utm_medium=editorial-email&utm_source=authorletters&utm_campaign=protocols.

As the corresponding author, your ORCID iD is verified in the submission system and will appear in the published article. PLOS supports the use of ORCID, and we encourage all coauthors to register for an ORCID iD and use it as well. Please encourage your coauthors to verify their ORCID iD within the submission system before final acceptance, as unverified ORCID iDs will not appear in the published article. *Only*  the individual author can complete the verification step; PLOS staff *cannot*  verify ORCID iDs on behalf of authors.

We look forward to receiving your revised manuscript.

Kind regards,

Wanli Zang, Ph.D.

Guest Editor

PLOS One

Journal Requirements:

When submitting your revision, we need you to address these additional requirements.

1.Please ensure that your manuscript meets PLOS ONE's style requirements, including those for file naming. The PLOS ONE style templates can be found at

https://journals.plos.org/plosone/s/file?id=wjVg/PLOSOne_formatting_sample_main_body.pdf and

https://journals.plos.org/plosone/s/file?id=ba62/PLOSOne_formatting_sample_title_authors_affiliations.pdf

2. Thank you for stating the following financial disclosure:

“This study was funded by Universidad Francisco de Vitoria (UFV2026‑22).”

Please state what role the funders took in the study.  If the funders had no role, please state: "The funders had no role in study design, data collection and analysis, decision to publish, or preparation of the manuscript."

If this statement is not correct you must amend it as needed.

Please include this amended Role of Funder statement in your cover letter; we will change the online submission form on your behalf.

3. Thank you for stating the following financial disclosure:

“This study was funded by Universidad Francisco de Vitoria (UFV2026‑22).”

We note that one or more of the authors is affiliated with the funding organization, indicating the funder may have had some role in the design, data collection, analysis or preparation of your manuscript for publication; in other words, the funder played an indirect role through the participation of the co-authors. If the funding organization did not play a role in the study design, data collection and analysis, decision to publish, or preparation of the manuscript and only provided financial support in the form of authors' salaries and/or research materials, please do the following:

1. Review your statements relating to the author contributions, and ensure you have specifically and accurately indicated the role(s) that these authors had in your study. These amendments should be made in the online form.

2. Confirm in your cover letter that you agree with the following statement, and we will change the online submission form on your behalf:

“The funder provided support in the form of salaries for authors [insert relevant initials], but did not have any additional role in the study design, data collection and analysis, decision to publish, or preparation of the manuscript. The specific roles of these authors are articulated in the ‘author contributions’ section.

4. Your ethics statement should only appear in the Methods section of your manuscript. If your ethics statement is written in any section besides the Methods, please move it to the Methods section and delete it from any other section. Please ensure that your ethics statement is included in your manuscript, as the ethics statement entered into the online submission form will not be published alongside your manuscript.

5. Please include captions for your Supporting Information files at the end of your manuscript, and update any in-text citations to match accordingly. Please see our Supporting Information guidelines for more information: http://journals.plos.org/plosone/s/supporting-information.

6. Please upload a copy of your study protocol that was approved by your ethics committee/IRB as a Supporting Information file. By the study protocol, we mean the complete and detailed plan for the conduct and analysis of the trial approved by the ethics committee/IRB. Please send this in the original language. If this is in a language other than English, please also provide a translation. [https://journals.plos.org/plosone/s/submission-guidelines#loc-guidelines-for-specific-study-types.

7. If the reviewer comments include a recommendation to cite specific previously published works, please review and evaluate these publications to determine whether they are relevant and should be cited. There is no requirement to cite these works unless the editor has indicated otherwise.

[Note: HTML markup is below. Please do not edit.]

Reviewers' comments:

Reviewer's Responses to Questions

**Comments to the Author**

1. Does the manuscript provide a valid rationale for the proposed study, with clearly identified and justified research questions?

The research question outlined is expected to address a valid academic problem or topic and contribute to the base of knowledge in the field.

Reviewer #1: Yes

Reviewer #2: Yes

**********

2. Is the protocol technically sound and planned in a manner that will lead to a meaningful outcome and allow testing the stated hypotheses?

The manuscript should describe the methods in sufficient detail to prevent undisclosed flexibility in the experimental procedure or analysis pipeline, including sufficient outcome-neutral conditions (e.g. necessary controls, absence of floor or ceiling effects) to test the proposed hypotheses and a statistical power analysis where applicable. As there may be aspects of the methodology and analysis which can only be refined once the work is undertaken, authors should outline potential assumptions and explicitly describe what aspects of the proposed analyses, if any, are exploratory.

Reviewer #1: Yes

Reviewer #2: Partly

**********

3. Is the methodology feasible and described in sufficient detail to allow the work to be replicable?

Descriptions of methods and materials in the protocol should be reported in sufficient detail for another researcher to reproduce all experiments and analyses. The protocol should describe the appropriate controls, sample size calculations, and replication needed to ensure that the data are robust and reproducible.

Reviewer #1: Yes

Reviewer #2: Yes

**********

4. Have the authors described where all data underlying the findings will be made available when the study is complete?

The PLOS Data policy requires authors to make all data underlying the findings described in their manuscript fully available without restriction, with rare exception, at the time of publication. The data should be provided as part of the manuscript or its supporting information, or deposited to a public repository. For example, in addition to summary statistics, the data points behind means, medians and variance measures should be available. If there are restrictions on publicly sharing data—e.g. participant privacy or use of data from a third party—those must be specified.

Reviewer #1: Yes

Reviewer #2: No

**********

5. Is the manuscript presented in an intelligible fashion and written in standard English?

PLOS ONE does not copyedit accepted manuscripts, so the language in submitted articles must be clear, correct, and unambiguous. Any typographical or grammatical errors should be corrected at revision, so please note any specific errors here.

Reviewer #1: Yes

Reviewer #2: Yes

**********

6. Review Comments to the Author

Please use the space provided to explain your answers to the questions above and, if applicable, provide comments about issues authors must address before this protocol can be accepted for publication. You may also include additional comments for the author, including concerns about research or publication ethics.

You may also provide optional suggestions and comments to authors that they might find helpful in planning their study.

(Please upload your review as an attachment if it exceeds 20,000 characters)

Reviewer #1: This study protocol describes a randomized controlled trial designed to evaluate the effectiveness of a psychoeducational intervention based on Self-Determination Theory and using functionality oriented body image strategies. The aim is to enhance autonomous motivation for exercise and improve psychological well-being among female university students in Health Sciences.

The supplemental protocol and the text presented study design and analysis were written consistently.

The design is a simple parallel procedure randomly allocated (1:1) to an experimental group or a wait-list control group following baseline assessment. The primary outcome is autonomous motivation for exercise, assessed using the Behavioral Regulation in Exercise Questionnaire-3. The are approximately 4 secondary outcomes.

The study design and analysis procedures were well thought out and well constructed with a power driven sample size section. The statistical analysis section is presented clearly. Analyses will follow the intention-to-treat principle, complemented by per-protocol analyses to examine the robustness and consistency of findings. The analysis is appropriate and quite routine for this type of endeavor. Specifically linear mixed-effects models will be fitted. These models will be estimated using the Restricted Maximum Likelihood (REML) approach. Fixed effects will include group (experimental vs. control), time (T0, T1, T2), and the group × time interaction. Random intercepts for participants will be included to account for intra individual variability. This modeling approach appropriately handles correlated repeated measures data and partial missing observations. The timeline as noted in Figure 1 is reasonable.

The data management section appears complete with a quality control procedures. In that section is noted that missing data will be handled under an intention-to-treat framework. For all primary and secondary continuous outcomes, missing values will be addressed using multiple imputation as described.

The investigators note the limitations and in particular the single institution setting challenging the generalizability of the results. However, for the most part it appears well designed with doable implementation.

1. On a minor note, although missing data is discussed there should also be a few words discussing what retention procedures will be used to minimize the amount of missing data during the study.

2. At the end of the Statistical section the discussion of the mediation analysis is a bit too vague and should be detailed further. What models specifically will apply in this context?

Reviewer #2: The authors have submitted a protocol for a randomized controlled trial for an SDT-based intervention to help reduce physical inactivity, with multiple secondary outcomes. They have selected outcomes that measure SDT mechanisms and have included behavioral outcomes as well. This is a well thought out study with viable implementation. I have noted some areas that I believe will help to improve the strength of the trial and impact of their hypothesized findings.

1. The authors wrote, “Students who are interested may voluntarily access the link, review detailed information about the study, and complete the eligibility screening. Only those who self‑enroll and meet the inclusion criteria will be asked to provide written informed consent prior to participation.” Does this mean there is not compensation? How are participants compensated? To me this sounds like a potential major flaw in the design. If all participants self-select into a trial about motivation, this seems to be contributing problematic bias. Secondly, if students are interested and motivated to participate, then they are allocated to the waitlist, this can ultimately overestimate the effects of the study. Waitlist controls are already known to overestimate trial effects due to participant dissatisfaction with their allocation and subsequent over reporting of problems/amotivation. Please rethink this and how to manage such biases and potential overestimation of the outcome.

2. The statistical analysis section is a major weakness of the protocol but can be easily strengthened. There is a good deal of information missing from the analysis plan. However, this can be cumbersome for a protocol paper. So, I recommend the authors create a separate Statistical Analysis Plan (SAP) and include this as a supplement. Stevens et al. (2023) provides a very nice SAP template that will help the authors fill in the missing information at the most recent state-of-the-art for trial reporting.

https://www.sciencedirect.com/science/article/pii/S2451865423000467

3. I recommend choosing a single endpoint for the primary analysis and include the other timepoint in a secondary analysis. Also note all timepoints in secondary analyses that will be tested. In, the reporting of the results I also recommend including a table with all measurement means and SDs for all timepoints to aid in meta-analyses.

4. State the primary outcome in terms of domain, measure, metric, and timepoint. You have domain and measure, but you are missing the metric (within group change, between-group endpoint) and the primary timepoint (at six-months post randomization or at treatment completion). For example, “The primary outcome will be endpoint motivation toward physical activity, assessed using the Behavioral Regulation in Exercise Questionnaire–3, at six-months post randomization.”

5. MI is no longer the preferred method of handling missing data. Mixed-model repeated measures (MMRM) is preferred for continuous outcomes, and GLMM for count/categorical. Since you have multiple timepoints, I highly recommend describing you primary analysis as MMRM, and then of course planning the analysis as such. This can also account for missingness. MI is unnecessary if using MMRM. The authors report to using linear mixed-effects models. I recommend you call this MMRM and discuss how missingness handled. R has mmrm and emmeans packages.

A detailed example:

The primary efficacy analysis used a mixed model for repeated measures (MMRM) including all post-randomization assessments of the primary outcome. Fixed effects included treatment group, assessment time, treatment-by-time interaction, and baseline outcome score. An unstructured covariance matrix was specified to model within-participant correlations across repeated measurements. Least-squares mean differences between groups at the primary endpoint were estimated from the model with corresponding 95% confidence intervals and p-values. This likelihood-based approach uses all available observations without imputing missing outcome data and provides valid inference under a missing-at-random assumption.

Mixed models for repeated measures (MMRM) use likelihood-based estimation that incorporates all observed outcome data from each participant. Participants contribute information up to the point at which data are available, and parameter estimates are obtained by maximizing the likelihood of the observed data. Under the assumption that data are Missing At Random (MAR)—that is, the probability of missingness depends only on observed variables included in the model and not on unobserved outcomes—the resulting estimates are unbiased and statistically efficient.

6. The authors wrote, "The allocation sequence will be generated by a researcher using a computerized random number generator to ensure concealment." This statement does not describe allocation concealment. Please describe how the allocation sequence was concealed until participants were enrolled and assigned to interventions.

7. There is a good deal of redundancy for blinding, randomization, and allocation in various subsections. I recommend making a subheading, "Randomization, Blinding, and Allocation," and just mention everything once in this section. Also, mention how blinding is conducted at: Provider, participant, outcome assessor (different if clinician rated), researcher, data analyst levels.

8. Create a subheading for the Wait-list Control.

9. I recommend a sensitivity analysis that accounts for fidelity issues if present.

10. There is also redundancy with noting of the study timepoints that can be streamlined.

11. How are the authors managing deviations from the protocol? They mention fidelity measures which is great, but they do not mention what they will do about contaminant care or what is allowed. Are participants allowed full access to mental health services, personal trainers, dieticians, etc.? Are these data collected and perhaps accounted for in sensitivity analyses if needed?

12. In the SPIRIT checklist the authors have several N/A designations. I recommend that the authors address each of these in the Protocol. If they are truly N/A, that state why this is the case. However, many of these should be addressed. For example, “Name and contact information for the trial sponsor.” If this was an unfunded study, state this.

**********

7. PLOS authors have the option to publish the peer review history of their article (what does this mean?). If published, this will include your full peer review and any attached files.

If you choose “no”, your identity will remain anonymous but your review may still be made public.

**Do you want your identity to be public for this peer review?**  For information about this choice, including consent withdrawal, please see our Privacy Policy.

Reviewer #1: No

Reviewer #2: **Yes:** Ethan Sahker

**********

[NOTE: If reviewer comments were submitted as an attachment file, they will be attached to this email and accessible via the submission site. Please log into your account, locate the manuscript record, and check for the action link "View Attachments". If this link does not appear, there are no attachment files.]

To ensure your figures meet our technical requirements, please review our figure guidelines: https://journals.plos.org/plosone/s/figures

You may also use PLOS’s free figure tool, NAAS, to help you prepare publication quality figures: https://journals.plos.org/plosone/s/figures#loc-tools-for-figure-preparation.

NAAS will assess whether your figures meet our technical requirements by comparing each figure against our figure specifications.

# Author comment: Author response to Decision Letter 1

**DOI:** 10.1371/journal.pone.0355374.r002

2 Jul 2026

We thank both Reviewers for their careful and constructive evaluation of our manuscript. We have revised the protocol accordingly and have also prepared a separate Statistical Analysis Plan (SAP v1.1, Supplementary File S3), following the template proposed by Stevens et al. (2023). Below we respond point-by-point to all comments. References to “SAP” denote sections of the Statistical Analysis Plan. Page and line numbers refer to the revised manuscript with tracked changes visible.

REVIEWER 1

We appreciate the reviewer’s positive evaluation of the study rationale, design, sample size justification, data management procedures, and planned analyses.

Comment 1 — Retention procedures

“…although missing data is discussed there should also be a few words discussing what retention procedures will be used to minimize the amount of missing data during the study.”

Response. Retention procedures have now been explicitly described. The protocol now states that periodic email reminders, advance notification of sessions and assessment points, systematic attendance monitoring, and reminders regarding the importance of continued participation will be implemented to minimise attrition and missing data. Additional details are provided in the SAP retention section.

Changes made: Manuscript page 7, lines 152–159; SAP 9.1.

Comment 2 — Mediation analysis too vague

“…the discussion of the mediation analysis is a bit too vague and should be detailed further. What models specifically will apply in this context?”

Response. The mediation analysis has been fully specified. The manuscript now states that mediation will be examined using a half-longitudinal model, with group allocation as the independent variable, T0–T1 change in autonomous motivation (BREQ-3 Relative Autonomy Index) as the mediator, and T1–T2 changes in physical activity (IPAQ-SF), positive body image (BAS-2), and eating disorder risk (EAT-26) as outcomes. The primary specification is a single-level structural equation model (SEM) implemented in lavaan, with bias-corrected bootstrap confidence intervals used to estimate indirect effects. In addition, a multilevel SEM accounting for partially nested intervention delivery subgroups has been pre-specified as a sensitivity analysis. Full methodological details are provided in the Statistical Analysis Plan (Supporting Information S3).

Changes made: Manuscript page 21, lines 464–471; SAP 8.4.

REVIEWER 2

We thank Reviewer 2 for the detailed methodological and statistical recommendations, which have helped to strengthen both the protocol and the Statistical Analysis Plan.

Comment 1 — Self-selection, wait-list overestimation, and compensation

“…If all participants self-select into a trial about motivation, this seems to be contributing problematic bias… waitlist controls are already known to overestimate trial effects… Please rethink this and how to manage such biases and potential overestimation…”

Response. The protocol now explicitly states that participants will not receive financial compensation. Participants allocated to the wait-list condition will receive delayed access to the complete intervention following completion of the follow-up assessment.

To address potential volunteer self-selection bias, the revised protocol clarifies that recruitment follows a census invitation approach in which all eligible students are contacted. Response rates will be reported, respondents will be compared against the eligible invited population using available administrative characteristics, and post-stratification/inverse-probability-of-response weighting sensitivity analyses will be conducted. The SAP additionally specifies ITT, per-protocol, and CACE analyses to evaluate the potential influence of self-selection and treatment adherence. The discussion section has also been expanded to explicitly acknowledge the possibility of effect overestimation associated with wait-list control designs.

Changes made: Manuscript page 7, lines 163–167; page 28, lines 616–623; SAP 9.2–9.3

Comment 2 — Provide a separate SAP (Stevens et al. template)

“…I recommend the authors create a separate Statistical Analysis Plan (SAP) and include this as a supplement. Stevens et al. (2023) provides a very nice SAP template…”

Response. A dedicated Statistical Analysis Plan (SAP v1.1) has been developed and included as Supplementary File S3. The SAP follows the template proposed by Stevens et al. (2023) and provides detailed specifications regarding estimands, outcomes, primary and secondary analyses, handling of missing data, mediation analyses, sensitivity analyses, and reporting procedures.

Changes made: Manuscript page 21, lines 477–478; Supplementary File S3 (SAP).

Comment 3 — Single primary endpoint, secondary timepoints, and a means/SD table

“…choosing a single endpoint for the primary analysis… note all timepoints in secondary analyses… include a table with all measurement means and SDs for all timepoints…”

Response. A single primary endpoint has now been defined. The primary analysis focuses on autonomous motivation (BREQ-3 Relative Autonomy Index) at post-intervention (T1). Analyses at T2 are explicitly defined as secondary analyses evaluating maintenance of intervention effects. A shell table reporting means and standard deviations for all outcomes and timepoints has also been incorporated into the SAP.

Changes made: Manuscript page 21, lines 464–467; SAP 4, 11 and 12.

Comment 4 — Primary outcome by domain, measure, metric, and timepoint

“State the primary outcome in terms of domain, measure, metric, and timepoint…”

Response. The primary outcome is now explicitly defined as follows: domain = autonomous motivation toward physical activity; measure = BREQ-3 Relative Autonomy Index (RAI); metric = between-group adjusted least-squares mean difference; primary timepoint = post-intervention (T1). (administration procude).

Changes made: SAP 3.2 and 4.

Comment 5 — MMRM instead of multiple imputation; GLMM for count/categorical

“MI is no longer the preferred method… MMRM is preferred for continuous outcomes, and GLMM for count/categorical… MI is unnecessary if using MMRM.”

Response. The manuscript and SAP have been revised accordingly. Multiple imputation has been removed as the primary strategy for handling missing data. The primary analysis is now specified as a Mixed Model for Repeated Measures (MMRM) under a Missing At Random (MAR) assumption, incorporating all observed data without imputation. For outcomes that do not satisfy distributional assumptions, appropriate generalized mixed models are specified.

Changes made: Manuscript page 19, lines 410–416; SAP 8.2, 8.3 and 9.1.

Comment 6 — Allocation concealment

“…This statement does not describe allocation concealment. Please describe how the allocation sequence was concealed until participants were enrolled and assigned…”

Response. Allocation concealment procedures have now been described in detail. The allocation sequence will be generated by an independent researcher not involved in recruitment, intervention delivery, or outcome assessment. The sequence will remain inaccessible to recruitment staff and will be revealed only after confirmation of eligibility and completion of baseline assessment. The protocol further specifies the use of variable block sizes and includes a pre-specified Berger–Exner assessment of allocation-selection bias.

Changes made: Manuscript page 10, lines 233–257; SAP 6.

Comment 7 — Consolidate Randomization, Blinding, and Allocation; blinding by level

“…make a subheading, ‘Randomization, Blinding, and Allocation,’ and mention everything once… mention how blinding is conducted at: Provider, participant, outcome assessor, researcher, data analyst levels.”

Response. Information regarding randomization, allocation concealment, and blinding has been consolidated into a single subsection entitled Randomization, Blinding, and Allocation. Blinding procedures are now explicitly described for participants, intervention providers, researchers, and data analysts.

Changes made: Manuscript page 10, lines 233–257; SAP 6.

Comment 8 — Subheading for the Wait-list Control

“Create a subheading for the Wait-list Control.”

Response. The intervention section has been reorganized to improve clarity and readability. In addition to incorporating a dedicated subheading for the Wait-list control condition, a corresponding Experimental intervention subheading has also been introduced. This structure allows both study conditions to be presented consistently and facilitates identification of the intervention and comparator arms throughout the protocol.

Changes made: Experimental intervention: line 270; Wait-list control condition: line 333.

Comment 9 — Sensitivity analysis for fidelity

“I recommend a sensitivity analysis that accounts for fidelity issues if present.”

Response. Fidelity-related sensitivity analyses have been pre-specified. In addition to the primary ITT analysis, fidelity-adjusted, adherence-based, and per-protocol analyses will be conducted to evaluate the robustness of findings in relation to intervention delivery and participant attendance. Further details are provided in the SAP.

Changes made: Manuscript page 20, lines 428–430; SAP 9.2.

Comment 10 — Redundancy in study timepoints

“There is also redundancy with noting of the study timepoints that can be streamlined.”

Response. Descriptions of T0, T1 and T2 have been reviewed throughout the manuscript and streamlined where possible to reduce redundancy while maintaining consistency across sections.

Changes made: Revisions throughout the manuscript.

Comment 11 — Deviations and concomitant/contaminant care

“…they do not mention what they will do about contaminant care or what is allowed… Are these data collected and perhaps accounted for in sensitivity analyses…?”

Response. The protocol now explicitly states that participants may continue accessing external services, including psychological support, personal training, dietary counselling, and other health-related services. These data will be collected at each assessment, summarised by treatment arm, evaluated for imbalance, and incorporated into sensitivity analyses as time-varying covariates where appropriate. Protocol deviations and co-interventions will be documented and reported.

Changes made: Manuscript page 16, lines 337–346; SAP 9.2 and 9.4.

Comment 12 — SPIRIT checklist N/A items and trial sponsor

“…address each of these in the Protocol. If they are truly N/A, state why… For example, ‘Name and contact information for the trial sponsor.’ If this was an unfunded study, state this.”

Response. The sponsor and funding source have now been explicitly reported in the protocol (Universidad Francisco de Vitoria; internal competitive funding call UFV2026-22). In addition, all SPIRIT checklist items previously marked as “N/A” were reviewed individually.

Several items that were previously marked as “N/A” have been completed following the reviewer’s recommendation. For those items that remain designated as “N/A”, we consider this designation appropriate given the characteristics of the present trial. Specifically, item 3d was retained as N/A because no steering committee, endpoint adjudication committee, or data monitoring committee has been established, as this is a single-centre, low-risk behavioural intervention coordinated directly by the principal investigators. Item 11 was retained as N/A because patients or members of the public were not involved in the design, conduct, reporting, or dissemination plans of this study, which was developed from previous empirical evidence and behavioural theory. Given the single-centre design, the behavioural nature of the intervention, and the minimal risks involved, oversight by an independent DMC is not considered necessary. Item 28b remains N/A because no interim analyses or stopping guidelines are planned owing to the low-risk nature and relatively short duration of the intervention. Item 32b also remains N/A because no biological specimens will be collected and no ancillary studies are planned; therefore, no additional consent beyond participation in the main trial is required.

Changes made: Funding section (manuscript page 1); revised SPIRIT checklist (S1 items 3d, 11, 28b, and 32b); SAP header

Attachment Submitted filename: 3. Response to Reviewers.pdf

# Peer review: Decision Letter 1

**DOI:** 10.1371/journal.pone.0355374.r003

**Authors:** Wanli Zang

10 Jul 2026

PONE-D-26-19756R1

Study protocol for a randomized controlled trial of a self-determination theory–based psychoeducational intervention to enhance autonomous motivation for exercise in female university students

PLOS One

Dear Dr. Ruiz-Bravo,

Thank you for submitting your manuscript to PLOS ONE. After careful consideration, we feel that it has merit but does not fully meet PLOS ONE’s publication criteria as it currently stands. Therefore, we invite you to submit a revised version of the manuscript that addresses the points raised during the review process.

Please submit your revised manuscript by Aug 24 2026 11:59PM. If you will need more time than this to complete your revisions, please reply to this message or contact the journal office at plosone@plos.org. When you're ready to submit your revision, log on to https://www.editorialmanager.com/pone/ and select the 'Submissions Needing Revision' folder to locate your manuscript file.

Please include the following items when submitting your revised manuscript:

- A letter that responds to each point raised by the academic editor and reviewer(s). You should upload this letter as a separate file labeled 'Response to Reviewers'.
- A marked-up copy of your manuscript that highlights changes made to the original version. You should upload this as a separate file labeled 'Revised Manuscript with Track Changes'.
- An unmarked version of your revised paper without tracked changes. You should upload this as a separate file labeled 'Manuscript'.

If you would like to make changes to your financial disclosure, please include your updated statement in your cover letter. Guidelines for resubmitting your figure files are available below the reviewer comments at the end of this letter.

If applicable, we recommend that you deposit your laboratory protocols in protocols.io to enhance the reproducibility of your results. Protocols.io assigns your protocol its own identifier (DOI) so that it can be cited independently in the future. For instructions see: https://journals.plos.org/plosone/s/submission-guidelines#loc-laboratory-protocols. Additionally, PLOS ONE offers an option for publishing peer-reviewed Lab Protocol articles, which describe protocols hosted on protocols.io. Read more information on sharing protocols at https://plos.org/protocols?utm_medium=editorial-email&utm_source=authorletters&utm_campaign=protocols.

As the corresponding author, your ORCID iD is verified in the submission system and will appear in the published article. PLOS supports the use of ORCID, and we encourage all coauthors to register for an ORCID iD and use it as well. Please encourage your coauthors to verify their ORCID iD within the submission system before final acceptance, as unverified ORCID iDs will not appear in the published article. *Only*  the individual author can complete the verification step; PLOS staff *cannot*  verify ORCID iDs on behalf of authors.

We look forward to receiving your revised manuscript.

Kind regards,

Wanli Zang, Ph.D.

Guest Editor

PLOS One

Journal Requirements:

1. If the reviewer comments include a recommendation to cite specific previously published works, please review and evaluate these publications to determine whether they are relevant and should be cited. There is no requirement to cite these works unless the editor has indicated otherwise.

2. Please review your reference list to ensure that it is complete and correct. If you have cited papers that have been retracted, please include the rationale for doing so in the manuscript text, or remove these references and replace them with relevant current references. Any changes to the reference list should be mentioned in the rebuttal letter that accompanies your revised manuscript. If you need to cite a retracted article, indicate the article’s retracted status in the References list and also include a citation and full reference for the retraction notice.

[Note: HTML markup is below. Please do not edit.]

Reviewers' comments:

Reviewer's Responses to Questions

**Comments to the Author**

1. Does the manuscript provide a valid rationale for the proposed study, with clearly identified and justified research questions?

The research question outlined is expected to address a valid academic problem or topic and contribute to the base of knowledge in the field.

Reviewer #1: Yes

Reviewer #2: Yes

**********

2. Is the protocol technically sound and planned in a manner that will lead to a meaningful outcome and allow testing the stated hypotheses?

The manuscript should describe the methods in sufficient detail to prevent undisclosed flexibility in the experimental procedure or analysis pipeline, including sufficient outcome-neutral conditions (e.g. necessary controls, absence of floor or ceiling effects) to test the proposed hypotheses and a statistical power analysis where applicable. As there may be aspects of the methodology and analysis which can only be refined once the work is undertaken, authors should outline potential assumptions and explicitly describe what aspects of the proposed analyses, if any, are exploratory.

Reviewer #1: Yes

Reviewer #2: Yes

**********

3. Is the methodology feasible and described in sufficient detail to allow the work to be replicable?

Descriptions of methods and materials in the protocol should be reported in sufficient detail for another researcher to reproduce all experiments and analyses. The protocol should describe the appropriate controls, sample size calculations, and replication needed to ensure that the data are robust and reproducible.

Reviewer #1: Yes

Reviewer #2: No

**********

4. Have the authors described where all data underlying the findings will be made available when the study is complete?

The PLOS Data policy requires authors to make all data underlying the findings described in their manuscript fully available without restriction, with rare exception, at the time of publication. The data should be provided as part of the manuscript or its supporting information, or deposited to a public repository. For example, in addition to summary statistics, the data points behind means, medians and variance measures should be available. If there are restrictions on publicly sharing data—e.g. participant privacy or use of data from a third party—those must be specified.

Reviewer #1: Yes

Reviewer #2: Yes

**********

5. Is the manuscript presented in an intelligible fashion and written in standard English?

PLOS ONE does not copyedit accepted manuscripts, so the language in submitted articles must be clear, correct, and unambiguous. Any typographical or grammatical errors should be corrected at revision, so please note any specific errors here.

Reviewer #1: Yes

Reviewer #2: Yes

**********

6. Review Comments to the Author

Please use the space provided to explain your answers to the questions above and, if applicable, provide comments about issues authors must address before this protocol can be accepted for publication. You may also include additional comments for the author, including concerns about research or publication ethics.

You may also provide optional suggestions and comments to authors that they might find helpful in planning their study.

(Please upload your review as an attachment if it exceeds 20,000 characters)

Reviewer #1: Comments have been addressed and revisions noted.

XXXXXXXXXXXXXXXXXXXXXXXXXXXXXXXXXXXXXXXXXXXXXXXXXXXXXXXXXXXXXXXXXXXXXXXXXXXXXXXXXXXXXXXXXXXXXXXXXXXXXXXXXXXXXXXXXXXXXXXXXXXXXXXXXXXXXXXXXXXXXXXXXXXXXXXXXXXXXXXX

Reviewer #2: The authors have been very responsive and I think these changes will really help reduce risks of bias and demonstrate a robust study procedure. However, one change they made is now posing a potentially critical error.

Comment 5 — MMRM instead of multiple imputation; GLMM for count/categorical

Response.

The manuscript and SAP have been revised accordingly. Multiple imputation has

been removed as the primary strategy for handling missing data. The primary analysis is now

specified as a Mixed Model for Repeated Measures (MMRM) under a Missing At Random

(MAR) assumption, incorporating all observed data without imputation. For outcomes that do

not satisfy distributional assumptions, appropriate generalized mixed models are specified.

-----

I'm happy to see the move to MMRM, However, now that the authors have identified a primary timepoint as T1, MMRM will not work for the primary outcome analysis. If the primary outcome is T1, there will only be two measurements, and MMRM requires a minimum of 3 timepoints to work. MMRM would be optimal if the primary outcome was analyzed at T2. A standard mixed model approach does not account for missingness and so MI is more appropriate if T1 is the primary endpoint.

The authors will now need to either change the primary outcome endpoint to T2, or change back to standard mixed model with MI for the primary outcome (SAP and Methods). MMRM/GLMM will still be most appropriate for secondary outcomes at T2.

**********

7. PLOS authors have the option to publish the peer review history of their article (what does this mean?). If published, this will include your full peer review and any attached files.

If you choose “no”, your identity will remain anonymous but your review may still be made public.

**Do you want your identity to be public for this peer review?**  For information about this choice, including consent withdrawal, please see our Privacy Policy.

Reviewer #1: No

Reviewer #2: No

**********

[NOTE: If reviewer comments were submitted as an attachment file, they will be attached to this email and accessible via the submission site. Please log into your account, locate the manuscript record, and check for the action link "View Attachments". If this link does not appear, there are no attachment files.]

To ensure your figures meet our technical requirements, please review our figure guidelines: https://journals.plos.org/plosone/s/figures

You may also use PLOS’s free figure tool, NAAS, to help you prepare publication quality figures: https://journals.plos.org/plosone/s/figures#loc-tools-for-figure-preparation.

NAAS will assess whether your figures meet our technical requirements by comparing each figure against our figure specifications.

# Author comment: Author response to Decision Letter 2

**DOI:** 10.1371/journal.pone.0355374.r004

14 Jul 2026

We thank the Editor and both Reviewers for their careful evaluation of the revised manuscript. We are pleased that the reviewers considered the previous comments satisfactorily addressed and that the methodological revisions were considered to strengthen the study protocol. We have carefully considered the remaining comment and revised both the manuscript and the Statistical Analysis Plan (SAP) accordingly.

Reviewer 1

We thank the reviewer for the positive evaluation of the revised manuscript and for confirming that the previous comments have been satisfactorily addressed.

Reviewer 2

Comment 5 — MMRM instead of multiple imputation; GLMM for count/categorical

“The manuscript and SAP have been revised accordingly. Multiple imputation has

been removed as the primary strategy for handling missing data. The primary analysis is now specified as a Mixed Model for Repeated Measures (MMRM) under a Missing At Random (MAR) assumption, incorporating all observed data without imputation. For outcomes that do not satisfy distributional assumptions, appropriate generalized mixed models are specified.

I'm happy to see the move to MMRM, However, now that the authors have identified a primary timepoint as T1, MMRM will not work for the primary outcome analysis. If the primary outcome is T1, there will only be two measurements, and MMRM requires a minimum of 3 timepoints to work. MMRM would be optimal if the primary outcome was analyzed at T2. A standard mixed model approach does not account for missingness and so MI is more appropriate if T1 is the primary endpoint.The authors will now need to either change the primary outcome endpoint to T2, or change back to standard mixed model with MI for the primary outcome (SAP and Methods). MMRM/GLMM will still be most appropriate for secondary outcomes at T2.”

Response. We thank the Reviewer for this constructive methodological critique. Following the Reviewer’s recommendations, our team has carefully re-evaluated the core conceptual and investigational goals of this trial. The fundamental purpose of our Self-Determination Theory (SDT)-based psychoeducational program is to foster deep internalization of exercise motives and sustainable behavioral changes. Therefore, from a clinical and behavioral perspective, the long-term maintenance of autonomous motivation at the 6-month follow-up (T2) represents our primary endpoint.

This refinement establishes a highly coherent framework:

• Methodological Soundness: By setting T2 as the primary endpoint, the MMRM now evaluates the full evaluation trajectory (T0 as a baseline covariate, T1 as an intermediate secondary milestone, and T2 as the primary endpoint). This satisfies the minimum requirement of 3 timepoints, mathematically validating the MMRM structure and completely preserving its capacity to handle missing data under MAR without requiring prior multiple imputation.

• Statistical Power Realignment: We have updated our simulation-based power script in R to align with this change.

We believe this adjustment has significantly strengthened the transparency, reproducibility, and mathematical internal validity of our protocol.

Changes made:

Manuscript

-Abstract: page 5, lines 36 and 38-39

-Sample Size: page 8, lines193-194 and 204

-Measures: Primary outcomes: page 15, lines 322

- Statistical Analysis: page 18, lines: 396–398 and 418).

SAP:

3.1, 3.2, 4, 5.2, 5.4, 5.5, 8.2 and 12

Attachment Submitted filename: 3. Response to Reviewers_2.docx

# Peer review: Decision Letter 2

**DOI:** 10.1371/journal.pone.0355374.r005

**Authors:** Wanli Zang

21 Jul 2026

Study protocol for a randomized controlled trial of a self-determination theory–based psychoeducational intervention to enhance autonomous motivation for exercise in female university students

PONE-D-26-19756R2

Dear Dr. Ruiz-Bravo,

We’re pleased to inform you that your manuscript has been judged scientifically suitable for publication and will be formally accepted for publication once it meets all outstanding technical requirements.

Within one week, you’ll receive an e-mail detailing the required amendments. When these have been addressed, you’ll receive a formal acceptance letter and your manuscript will be scheduled for publication.

An invoice will be generated when your article is formally accepted. Please note, if your institution has a publishing partnership with PLOS and your article meets the relevant criteria, all or part of your publication costs will be covered. Please make sure your user information is up-to-date by logging into Editorial Manager at Editorial Manager® and clicking the ‘Update My Information' link at the top of the page. For questions related to billing, please contact billing support.

If your institution or institutions have a press office, please notify them about your upcoming paper to help maximize its impact. If they’ll be preparing press materials, please inform our press team as soon as possible -- no later than 48 hours after receiving the formal acceptance. Your manuscript will remain under strict press embargo until 2 pm Eastern Time on the date of publication. For more information, please contact onepress@plos.org.

Kind regards,

Wanli Zang, Ph.D.

Guest Editor

PLOS One

Additional Editor Comments (optional):

Reviewers' comments:

Reviewer's Responses to Questions

**Comments to the Author**

1. Does the manuscript provide a valid rationale for the proposed study, with clearly identified and justified research questions?

The research question outlined is expected to address a valid academic problem or topic and contribute to the base of knowledge in the field.

Reviewer #1: Yes

**********

2. Is the protocol technically sound and planned in a manner that will lead to a meaningful outcome and allow testing the stated hypotheses?

The manuscript should describe the methods in sufficient detail to prevent undisclosed flexibility in the experimental procedure or analysis pipeline, including sufficient outcome-neutral conditions (e.g. necessary controls, absence of floor or ceiling effects) to test the proposed hypotheses and a statistical power analysis where applicable. As there may be aspects of the methodology and analysis which can only be refined once the work is undertaken, authors should outline potential assumptions and explicitly describe what aspects of the proposed analyses, if any, are exploratory.

Reviewer #1: Yes

**********

3. Is the methodology feasible and described in sufficient detail to allow the work to be replicable?

Descriptions of methods and materials in the protocol should be reported in sufficient detail for another researcher to reproduce all experiments and analyses. The protocol should describe the appropriate controls, sample size calculations, and replication needed to ensure that the data are robust and reproducible.

Reviewer #1: Yes

**********

4. Have the authors described where all data underlying the findings will be made available when the study is complete?

The PLOS Data policy requires authors to make all data underlying the findings described in their manuscript fully available without restriction, with rare exception, at the time of publication. The data should be provided as part of the manuscript or its supporting information, or deposited to a public repository. For example, in addition to summary statistics, the data points behind means, medians and variance measures should be available. If there are restrictions on publicly sharing data—e.g. participant privacy or use of data from a third party—those must be specified.

Reviewer #1: Yes

**********

5. Is the manuscript presented in an intelligible fashion and written in standard English?

PLOS ONE does not copyedit accepted manuscripts, so the language in submitted articles must be clear, correct, and unambiguous. Any typographical or grammatical errors should be corrected at revision, so please note any specific errors here.

Reviewer #1: Yes

**********

6. Review Comments to the Author

Please use the space provided to explain your answers to the questions above and, if applicable, provide comments about issues authors must address before this protocol can be accepted for publication. You may also include additional comments for the author, including concerns about research or publication ethics.

You may also provide optional suggestions and comments to authors that they might find helpful in planning their study.

(Please upload your review as an attachment if it exceeds 20,000 characters)

Reviewer #1: No Comment.

XXXXXXXXXXXXXXXXXXXXXXXXXXXXXXXXXXXXXXXXXXXXXXXXXXXXXXXXXXXXXXXXXXXXXXXXXXXXXXXXXXXXXXXXXXXXXXXXXXXXXXXXXXXXXXXXXXXXXXXXXXXXXXXXXXXXXXXXXXXXXXXXXXXXXXXXXXXXXXXXXXXXXXXXX

**********

7. PLOS authors have the option to publish the peer review history of their article (what does this mean?). If published, this will include your full peer review and any attached files.

If you choose “no”, your identity will remain anonymous but your review may still be made public.

**Do you want your identity to be public for this peer review?**  For information about this choice, including consent withdrawal, please see our Privacy Policy.

Reviewer #1: No

**********

# Editor report: Acceptance letter

**DOI:** 10.1371/journal.pone.0355374.r006

**Authors:** Wanli Zang

PONE-D-26-19756R2

PLOS One

Dear Dr. Ruiz-Bravo,

I'm pleased to inform you that your manuscript has been deemed suitable for publication in PLOS One. Congratulations! Your manuscript is now being handed over to our production team.

At this stage, our production department will prepare your paper for publication. This includes ensuring the following:

* All references, tables, and figures are properly cited

* All relevant supporting information is included in the manuscript submission,

* There are no issues that prevent the paper from being properly typeset

You will receive further instructions from the production team, including instructions on how to review your proof when it is ready. Please keep in mind that we are working through a large volume of accepted articles, so please give us a few days to review your paper and let you know the next and final steps.

Lastly, if your institution or institutions have a press office, please let them know about your upcoming paper now to help maximize its impact. If they'll be preparing press materials, please inform our press team within the next 48 hours. Your manuscript will remain under strict press embargo until 2 pm Eastern Time on the date of publication. For more information, please contact onepress@plos.org.

You will receive an invoice from PLOS for your publication fee after your manuscript has reached the completed accept phase. If you receive an email requesting payment before acceptance or for any other service, this may be a phishing scheme. Learn how to identify phishing emails and protect your accounts at https://explore.plos.org/phishing.

If we can help with anything else, please email us at customercare@plos.org.

Thank you for submitting your work to PLOS ONE and supporting open access.

Kind regards,

PLOS ONE Editorial Office Staff

on behalf of

Dr. Wanli Zang

Guest Editor

PLOS One
